# Analytical comparisons for solving the modified fractional Kawahara equation: application on numerical simulation of chemical signaling processes

**DOI:** 10.1038/s41598-026-57405-5

**Published:** 2026-06-11

**Authors:** Faten H. Damag, Mohammad Alshammari, Amin Saif, Adem Kiliçman, Hakeem A. Othman, Suliman Dawood

**Affiliations:** 1 Department of Mathematics, Faculty of Sciences, Ha’il University, Ha’il, 55473 Saudi Arabia; 2 Department of Computer Science, Faculty of Applied Sciences, Taiz University, Taiz 6803, Yemen; 3 Department of Mathematics, Faculty of Applied Sciences, Taiz University, Taiz, Yemen; 4 School of Mathematical Sciences, College of Computing, Informatics and Mathematics, Selangor, Malaysia; 5 Department of Mathematics, Albaydah University, Albaydah, Yemen; 6 Department of Mathematics, Faculty of Education, Hodeidah University, Hodeidah, Yemen

**Keywords:** Yang transform, Caputo Derivative operator, Residual power series, Engineering, Mathematics and computing, Physics

## Abstract

Fractional models are essential for describing nonlinear, memory dependent wave phenomena in complex media, yet solving high order fractional nonlinear PDEs such as the Modified Caputo Fractional Kawahara Equation (MCFKE) remains challenging. This work introduces the Yang Residual Power Series Method (Yang RPSM), which integrates the Yang transform with a residual-based power series expansion to generate efficient semi-analytical solutions. Stability and convergence of the iterative scheme are established. Numerical comparisons show that the Yang RPSM outperforms natural transform decomposition technique (NTDT) and homotopy analysis technique (HAT) in accuracy and computational behavior. Applications to intracellular Ca$^{2+}$ propagation further demonstrate that the MCFKE effectively captures memory effects, nonlinear wave steepening, and dispersion-driven attenuation. The Yang RPSM provides a reliable computational tool for high order fractional PDEs and highlights the MCFKE as a biologically meaningful model for anomalous, memory driven wave processes.

## Introduction

In recent years, the theory of partial fractional differential equations has emerged as an important field of mathematics, providing effective tools for modeling a wide range of systems across diverse scientific disciplines, engineering, including physics, medicine, fluid mechanics, electrical networks, groundwater dynamics, and polymer science^1–5^. A notable application lies in the study of shallow water wave equations, which are widely employed to describe phenomena such as shock waves, storm surges, and tsunami propagation. Within this context, the Kawahara and modified Kawahara equations form^6^ have been introduced to characterize solitary-wave propagation^7^. They are defined as follows:

1 $$\begin{aligned} \frac{\partial \pi (\theta _{1},\theta _{2})}{\partial \theta _{2}} +\pi (\theta _{1},\theta _{2}) \frac{\partial \pi (\theta _{1},\theta _{2})}{\partial \theta _{1}}+\frac{\partial ^{3} \pi (\theta _{1},\theta _{2})}{\partial \theta _{1}^{3}}-\frac{\partial ^{5} \pi (\theta _{1},\theta _{2})}{\partial \theta _{1}^{5}}=0. \end{aligned}$$

and

2 $$\begin{aligned} \frac{\partial \pi (\theta _{1},\theta _{2})}{\partial \theta _{2}} +\pi ^{2}(\theta _{1},\theta _{2}) \frac{\partial \pi (\theta _{1},\theta _{2})}{\partial \theta _{1}}+\lambda \frac{\partial ^{3} \pi (\theta _{1},\theta _{2})}{\partial \theta _{1}^{3}}-\lambda '\frac{\partial ^{5} \pi (\theta _{1},\theta _{2})}{\partial \theta _{1}^{5}}=0. \end{aligned}$$

The symmetry laws and generalized conservation properties of the Kawahara equations were studied in^8^. These equations have found numerous applications across mathematics and applied sciences^9–12^. The origins of fractional calculus can be traced back to 1695, with the development of fractional derivatives and integrals as generalizations of classical differentiation and integration to arbitrary (non-integer) orders. Among the earliest operators introduced in this field are the Riemann-Liouville and Caputo fractional differential operators^13^. Building upon these foundations, several researchers have proposed various fractional operators, including^14–16^. Fractional calculus theory has several applications in diverse scientific areas such as electromagnetism, wave propagation, heat transfer, robotics system classification, physics, mechanics, and viscoelasticity. Alsallami et al.^17^ introduced a time-fractional formulation of the Belousov-Zhabotinsky reaction model using both singular and non-singular kernels to capture memory-dependent chemical dynamics. Chauhan et al.^18^ introduced a Caputo-type fractional-order model for the transmission dynamics of chlamydia disease and studied the stability properties of the system. Chauhan et al.^19^ introduced a unique semi-analytic technique for solving fractional Fokker–Planck and Fornberg–Whitham equations with improved accuracy. One particularly important application is the MCFKE in shallow water waves, which is defined by

3 $$\begin{aligned} {}^{c}\mathcal {D}_{0,\theta _{2}}^{\eta }\pi (\theta _{1},\theta _{2})+\pi ^{2}(\theta _{1},\theta _{2})\frac{\partial \pi (\theta _{1},\theta _{2})}{\partial \theta _{1}}+\lambda \frac{\partial ^{3} \pi (\theta _{1},\theta _{2})}{\partial \theta _{1}^{3}}+\lambda '\frac{\partial ^{5} \pi (\theta _{1},\theta _{2})}{\partial \theta _{1}^{5}}=0,\,\,\,\,\,\,\,\,0<\eta \le 1 \end{aligned}$$

where ${}^{c}\mathcal {D}_{0,\theta _{2}}^{\eta }$ is the Caputo operator of order *η*, *λ >0* and *λ '<0* are real numbers. There exist several mathematical methods and efficient techniques for solving FPDEs. Examples include: the Aboodh residual power series method^20^ for solving the B-S differential equations, the modified expansion map method^21^, the S-G expansion technique applied to W-Z system models^22^, the expansion method^23^, the monotone iterative technique for reaction-diffusion equations^24^, the fractional Newton method^25^, the homotopy perturbation method^26^, the Laplace transform method^1^, the reproducing kernel Hilbert space method^27^, the homotopy analysis method^28^, the Laplace residual power series method^29^, and a modified Adams-Bashforth method^30^. Ali and Maneea^31^ introduced the optimal homotopy analysis method for constructing optical soliton solutions of time-fractional (1+1)-dimensional coupled nonlinear Schrödinger equations. Khirsariya et al.^32^ introduced a semi-analytic solution framework for the fractional modified Kawahara equation and examined the convergence and effectiveness of the proposed approach. Ali et al.^33^ introduced a semi-analytic technique to derive optical soliton solutions of the time-fractional *q*-deformed sinh-Gordon equation, emphasizing the influence of fractional-order parameters on soliton dynamics. Makwana and Khirsariya^34^ introduced a modified Adomian decomposition method to obtain analytical solutions for nonlinear fractional Kaup-Kupershmidt equations. Specifically for the MCFKE (3), several techniques have been developed, including the septic B-spline collocation method^35^, the HAT^36^, an innovative analytical method for solving the N-Wh-S equation^37^, the fixed-point theorem with HAT^38^, the Laplace Adomian decomposition method^39^, the NTDT^40^, the iterative Laplace transform method^41^, and the RPSM^42^. In intracellular biochemical environments, signaling molecules move through crowded and heterogeneous media, producing memory and anomalous transport effects. These behaviors correspond directly to the fractional, nonlinear, and dispersive terms in the MCFKE (3), making it a physically grounded model for chemical wave propagation in cells.

The present study is driven by the need to develop effective techniques for solving complex FPDEs, particularly the MCFKE (3), which models anomalous, dispersive, and memory-dependent behavior in chemical signaling processes. Although existing methods offer useful insights, many are limited in terms of accuracy, computational efficiency, or analytical tractability. To overcome these challenges, we introduce a novel hybrid approach, the Yang RPSM, aimed at generating approximate analytical solutions while demonstrating its capability in modeling significant biochemical phenomena, such as calcium ion (Ca$^{2+}$) wave propagation.

In this work, we formulate and analyze the Yang RPSM to obtain accurate semi-analytical solutions for the MCFKE (3), a higher-order nonlinear fractional model that captures memory-dependent and dispersive wave dynamics. The stability and convergence of the proposed method are rigorously established, and its accuracy is evaluated through comparisons with the NTDT and HAT techniques. Furthermore, the method is applied to intracellular Ca$^{2+}$ signaling to highlight the biological significance of the MCFKE model.

The paper is organized as follows: Section 1 presents the motivation for fractional modeling in chemical and intracellular signaling and introduces the MCFKE. Section 2 outlines the Yang Transform and the Residual Power Series Method, forming the basis of the Yang RPSM. Section 3 develops the Yang RPSM and derives the recursive coefficients for constructing the semi-analytical solution. Section 4 examines the stability and convergence of the method. Section 5 provides a biochemical interpretation of the model and discusses Ca$^{2+}$ simulations. Finally, Section 6 presents numerical results and compares the proposed method with existing approaches.

The novelty of the present work does not reside in a new formulation of the MCFKE itself, but rather in the analytical methodology employed for its solution. Specifically, the main contribution lies in coupling the Yang transform with the residual power series framework, which naturally induces a multiple fractional power series (MFPS) structure in the transform domain. This MFPS formulation leads to a novel recursive coefficient generation mechanism based on residual limits as the transform variable approaches zero.

Accordingly, the analytical treatment of the MCFKE serves as an application that demonstrates the effectiveness of this transform-induced recursive framework. Furthermore, the proposed Yang–RPSM introduces a coefficient construction mechanism that yields a multiple fractional power series in positive powers of the transform variable. This feature simplifies the computation of coefficients and enhances stability compared to existing approaches such as Laplace-based RPSM, NTDT, and HAT, which often rely on inverse-power expansions, decomposition schemes, or auxiliary convergence-control parameters.

## On Yang and Caputo operators

In this section, we first recall the definition of the Caputo derivative is used in the MCFKE (3) because it naturally models memory effects while allowing standard physical initial conditions. Its power law kernel captures the anomalous diffusion and history dependent behavior typical of intracellular Ca$^{2+}$ signaling. Let *π* be any mapping on $I\times [0,\infty )$, where *I* is an interval. The R-L operator,^43^, of $\pi (\theta _{1},\theta _{2})$ for order *η > 0* is given by

4 $$\begin{aligned} \mathcal {D}_{0,\theta _{2}}^{\eta }\pi (\theta _{1},\theta _{2})=\partial _{\theta _{2}}^{n}\mathcal {I}_{0,\theta _{2}}^{\eta }\pi (\theta _{1},\theta _{2})\,\,\,\,\,\,\,\,\, n-1<\eta <n \end{aligned}$$

where $\mathcal {I}_{0,\theta _{2}}^{\eta }$ is the R-L integral,^43^, of $\pi (\theta _{1},\theta _{2})$ of order *η* is given by

5 $$\begin{aligned} \mathcal {I}_{0,\theta _{2}}^{\eta }\pi (\theta _{1},\theta _{2})= \left\{ \begin{aligned}&\frac{1}{\Gamma (\eta )}\int _{0}^{\theta _{2}}(\theta _{2}-\mathcal {T})^{\eta -1} \pi (\theta _{1},\mathcal {T}) d\mathcal {T},\,\,\,\,\,\,\,\,\eta >0\\&\pi (\theta _{1},\theta _{2}),\,\,\,\,\,\,\,\,\eta =0.\\ \end{aligned} \right. \end{aligned}$$

The Caputo operator,^44^, for $\pi (\theta _{1},\theta _{2})$ of order *η* is defined by

6 $$\begin{aligned} {}^{c}\mathcal {D}_{0,\theta _{2}}^{\eta }\pi (\theta _{1},\theta _{2})= \left\{ \begin{aligned}&\mathcal {I}_{0,\theta _{2}}^{n-\eta } \partial ^{n}_{\theta _{2}} \pi (\theta _{1},\theta _{2}),\,\,\,\,\,\,\,\,n-1<\eta <n\\&\partial ^{\eta }_{\theta _{2}} \pi (\theta _{1},\theta _{2}),\,\,\,\,\,\,\,\,\eta \in \mathbb {N},\\ \end{aligned} \right. \end{aligned}$$

For $\theta _{2}\ge 0$ and for *n-1<η <n*, by^44^ we have ${}^{c}\mathcal {D}_{0,\theta _{2}}^{\eta }\mathcal {I}_{0,\theta _{2}}^{\eta }\pi (\theta _{1},\theta _{2})=\pi (\theta _{1},\theta _{2})$, ${}^{c}\mathcal {D}_{0,\theta _{2}}^{\eta } \theta _{2}^{\gamma }=\frac{\Gamma (\eta +2)}{\Gamma (\gamma -\eta +2)}\theta _{2}^{\gamma -\eta }$ and

$${\mathcal {I}}_{0,\theta _{2}}^{\eta } { ^{c}}{\mathcal {D}}_{0,\theta _{2}}^{\eta }\pi (\theta _{1},\theta _{2})=\pi (\theta _{1},\theta _{2})+\sum _{k=0}^{n-1}\frac{\theta _{2}^{k}}{k!} {\partial }^{k}_{\theta _{2}} \pi (\theta _{1},\theta _{2})\Big |_{\theta _{2}=0},$$

where *γ >-1*.

Let $\pi (\theta _{1},\theta _{2})$ be an order-exponential map *δ* on $I\times [0,\infty )$. The Yang transform $\mathbb {Y}_{\theta _{2}\upsilon }$,^45^, of $\pi (\theta _{1},\theta _{2})$ w.r.t $\theta _{2}$ is defined by

7 $$\begin{aligned} \pi ^{\mathbb {Y}}_{\theta _{2}}(\theta _{1},\upsilon ):=\mathbb {Y}_{\theta _{2}\upsilon }\pi (\theta _{1},\theta _{2})= \int _{0}^{\infty }e^{-\frac{\theta _{2}}{\upsilon }}\pi (\theta _{1},\theta _{2})d\theta _{2}\,\,\,\,\, \delta _{1}\le \upsilon \le \delta _{2}. \end{aligned}$$

Although the Yang transform is mathematically related to the classical Laplace transform through the relation $\mathbb {Y}[f(\theta _2),v] = \mathcal {L}[f(\theta _2),1/v],$ its use within the Residual Power Series Method leads to significant analytical and computational advantages. In particular, the Yang transform naturally generates series expansions in positive powers of the transform variable *v*, which aligns directly with the MFPS structure used in the Yang RPSM. This avoids inverse-power expansions that typically arise in the Laplace domain and simplifies the recursive determination of series coefficients. Moreover, fractional Caputo derivatives under the Yang transform yield compact algebraic expressions involving negative powers of *v*, allowing the residual conditions to be enforced through straightforward limits as *v → 0*. This feature significantly reduces algebraic complexity and improves numerical stability when constructing semi-analytical solutions of high-order fractional nonlinear equations such as the MCFKE. The inverse Yang transform $\mathbb {Y}_{\theta _{2}\upsilon }^{-1}$ of $\pi ^{\mathbb {Y}}_{\theta _{2}}(\theta _{1},\upsilon )$ w.r.t *υ* is given by

8 $$\begin{aligned} \mathbb {Y}_{\theta _{2}\upsilon }^{-1}\pi ^{\mathbb {Y}}_{\theta _{2}}(\theta _{1},\upsilon )=\pi (\theta _{1},\theta _{2}). \end{aligned}$$

Note that the Yang transform and its inverse are linear maps, that is,

1. $\mathbb {Y}_{\theta _{2}\upsilon }[c_{1}\pi (\theta _{1},\theta _{2})+c_{2}\rho (\theta _{1},\theta _{2})]=c_{1}\mathbb {Y}_{\theta _{2}\upsilon }\pi (\theta _{1},\theta _{2})+c_{2}\mathbb {Y}_{\theta _{2}\upsilon }\rho (\theta _{1},\theta _{2})$, where $c_{1}$ and $c_{2}$ are constants;
2. $\mathbb {Y}_{\theta _{2}\upsilon }^{-1}[c_{1}\mathbb {Y}_{\theta _{2}\upsilon }\pi (\theta _{1},\theta _{2})+c_{2}\mathbb {Y}_{\theta _{2}\upsilon }\rho (\theta _{1},\theta _{2})]=c_{1}\pi (\theta _{1},\theta _{2})+c_{2}\rho (\theta _{1},\theta _{2})$, where $c_{1}$ and $c_{2}$ are constants.

### Theorem 2.1

^45^ The Laplace transform of Caputo operator is given by

9 $$\begin{aligned} L_{\theta _{2}\upsilon }\left[ {}^{c}\mathcal {D}_{0,\theta _{2}}^{\eta }\pi (\theta _{1},\theta _{2}),\upsilon \right] = \upsilon ^{\eta }\pi ^{\mathbb {Y}}_{\theta _{2}}(\theta _{1},\upsilon )-\sum _{j=0}^{n-1}\upsilon ^{\eta -j-1}\partial ^{j}_{\theta _{2}}\pi (\theta _{1},\theta _{2})\Big |_{\theta _{2}=0} \,\,\, (n-1<\eta <n). \end{aligned}$$

To apply the Yang transform within the fractional framework, we first need to understand how the transform acts on the Caputo derivative. The following lemma provides an explicit Yang transform formula for the Caputo operator, which forms the foundation for constructing the Yang RPSM. Although the Yang transform is related to the Laplace transform via $\mathbb {Y}[f(t)](\upsilon )=\mathcal {L}[f(t)](1/\upsilon )$, the proposed Yang-RPSM is not a direct reformulation of Laplace RPSM or standard RPSM. The novelty is that the Yang transform induces an MFPS representation in the transform domain inpositive powers of *υ*. This representation is naturally compatible with the residual power series construction and leads to a different coefficient-extraction rule based on the limit *v→ 0*. As a result, the recursive computation of the fractional-series coefficients is algebraically simpler than in Laplace-domain residual schemes, which typically involve inverse-power expansions. In Laplace based residual formulations, the transformed solution typically produces inverse powers of the transform variable, which complicates recursive coefficient identification. In contrast, the Yang transform generates an MFPS expansion in positive powers of *υ* that matches the fractional-series structure used in the method, enabling coefficient extraction by a direct residual limit condition as $\upsilon \rightarrow 0$.

### Lemma 2.2

For any map *π*,

10 $$\begin{aligned} \mathbb {Y}_{\theta _{2}\upsilon }\left[ {}^{c}\mathcal {D}_{0,\theta _{2}}^{\eta }\pi (\theta _{1},\theta _{2})\right] = \upsilon ^{-\eta }\pi ^{\mathbb {Y}}_{\theta _{2}}(\theta _{1},\upsilon )-\sum _{j=0}^{n-1}\upsilon ^{j-\eta +1}\partial ^{j}_{\theta _{2}}\pi (\theta _{1},\theta _{2})\Big |_{\theta _{2}=0} \,\,\, (n-1<\eta <n). \end{aligned}$$

### Proof

Note that by Laplace-Yang property, we have

$$\begin{aligned} \mathbb {Y}_{\theta _{2}\upsilon }\left[ ^{c}\mathcal {D}_{0,\theta _{2}}^{\eta }\pi (\theta _{1},\theta _{2}),\upsilon \right] =L_{\theta _{2}\upsilon }\left[ ^{c}\mathcal {D}_{0,\theta _{2}}^{\eta }\pi (\theta _{1},\theta _{2}),\frac{1}{\upsilon }\right] . \end{aligned}$$

Hence

11 $$\begin{aligned} \begin{aligned}&\mathbb {Y}_{\theta _{2}\upsilon }\left[ {}^{c}\mathcal {D}_{0,\theta _{2}}^{\eta }\pi (\theta _{1},\theta _{2})\right] =\left( \frac{1}{\upsilon }\right) ^{\eta }\pi ^{\mathbb {Y}}_{\theta _{2}}(\theta _{1},\upsilon )-\sum _{j=0}^{n-1}\left( \frac{1}{\upsilon }\right) ^{\eta -j-1}\partial ^{j}_{\theta _{2}}\pi (\theta _{1},\theta _{2})\Big |_{\theta _{2}=0}\\&=\upsilon ^{-\eta }\pi ^{\mathbb {Y}}_{\theta _{2}}(\theta _{1},\upsilon )-\sum _{j=0}^{n-1}\upsilon ^{j-\eta +1}\partial ^{j}_{\theta _{2}}\pi (\theta _{1},\theta _{2})\Big |_{\theta _{2}=0}. \end{aligned} \end{aligned}$$

This is the desired. *□*

Since the Yang RPSM requires repeated application of fractional derivatives when building the series terms, it is necessary to generalize the Yang transform of the Caputo operator to higher orders. The next lemma extends Lemma above to the *kη* order case, enabling the recursive structure used later in the method.

### Definition 2.3

A function $\pi (\theta _1, \theta _2): I \times [0,\infty ) \rightarrow \mathbb {R}$ is called an order-exponential map of fractional order *η*, where $0 < \eta \le 1$, if it admits a multiple fractional power series (MFPS) expansion of the form

12 $$\begin{aligned} \pi (\theta _1, \theta _2) = \sum _{j=0}^{\infty } f_j(\theta _1)\,\theta _2^{j\eta }, \end{aligned}$$

where $f_j(\theta _1)$ are continuous functions on *I*, and the series converges for $\theta _2 \ge 0$.

### Lemma 2.4

Let $\pi :I\times [0,\infty )\rightarrow \mathbb {R}$ be a map on $I\times [0,\infty )$. Then

13 $$\begin{aligned} \mathbb {Y}_{\theta _{2}\upsilon }\left[ {}^{c}\mathcal {D}_{0,\theta _{2}}^{k\eta }\pi (\theta _{1},\theta _{2})\right] = \upsilon ^{-k\eta }\pi ^{\mathbb {Y}}_{\theta _{2}}(\theta _{1},\upsilon )-\sum _{j=0}^{k-1}\upsilon ^{(j-k)\eta +1}\,{}^{c}\mathcal {D}_{0,\theta _{2}}^{j\eta }\pi (\theta _{1},\theta _{2})\Big |_{\theta _{2}=0} \, (0<\eta \le 1). \end{aligned}$$

### Proof

We will prove (13) by using the induction. At *k=1*, since $0<\eta \le 1$ in (13), that is, *n=0* in Lemma above. Hence

14 $$\begin{aligned} \mathbb {Y}_{\theta _{2}\upsilon }\left[ {}^{c}\mathcal {D}_{0,\theta _{2}}^{\eta }\pi (\theta _{1},\theta _{2})\right] = \upsilon ^{-\eta }\pi ^{\mathbb {Y}}_{\theta _{2}}(\theta _{1},\upsilon )-\upsilon ^{-\eta +1}\pi (\theta _{1},0). \end{aligned}$$

It holds at *k=1*. If *k=2*, let $\rho (\theta _{1},\theta _{2})= {}^{c}\mathcal {D}_{0,\theta _{2}}^{\eta }\pi (\theta _{1},\theta _{2})$. By (14) above we have

15 $$\begin{aligned} \begin{aligned}&\mathbb {Y}_{\theta _{2}\upsilon }\left[ {}^{c}\mathcal {D}_{0,\theta _{2}}^{\eta }\rho (\theta _{1},\theta _{2})\right] = \upsilon ^{-\eta }\rho ^{\mathbb {Y}}_{\theta _{2}}(\theta _{1},\upsilon )-\upsilon ^{-\eta +1}\rho (\theta _{1},0)\\&= \upsilon ^{-\eta }\mathbb {Y}_{\theta _{2}\upsilon }{}^{c}\mathcal {D}_{0,\theta _{2}}^{\eta }\pi (\theta _{1},\theta _{2})-\upsilon ^{-\eta +1}\,\,{}^{c}\mathcal {D}_{0,\theta _{2}}^{\eta }\pi (\theta _{1},\theta _{2})\Big |_{\theta _{2}=0}\\&= \upsilon ^{-\eta }\left[ \upsilon ^{-\eta }\pi ^{\mathbb {Y}}_{\theta _{2}}(\theta _{1},\upsilon )-\upsilon ^{-\eta +1}\pi (\theta _{1},0)\right] -\upsilon ^{-\eta +1}\,\,{}^{c}\mathcal {D}_{0,\theta _{2}}^{\eta }\pi (\theta _{1},\theta _{2})\Big |_{\theta _{2}=0}\\&= \upsilon ^{-2\eta }\pi ^{\mathbb {Y}}_{\theta _{2}}(\theta _{1},\upsilon )-\upsilon ^{-2\eta +1}\pi (\theta _{1},0) -\upsilon ^{-\eta +1}\,\,{}^{c}\mathcal {D}_{0,\theta _{2}}^{\eta }\pi (\theta _{1},\theta _{2})\Big |_{\theta _{2}=0}. \end{aligned} \end{aligned}$$

It holds at *k=2*. Let (13) is true at *k=m*. Hence

16 $$\begin{aligned} \mathbb {Y}_{\theta _{2}\upsilon }\left[ {}^{c}\mathcal {D}_{0,\theta _{2}}^{-m\eta }\pi (\theta _{1},\theta _{2})\right] = \upsilon ^{-m\eta }\pi ^{\mathbb {Y}}_{\theta _{2}}(\theta _{1},\upsilon )-\sum _{j=0}^{m-1}\upsilon ^{(j-m)\eta +1}\,{}^{c}\mathcal {D}_{0,\theta _{2}}^{j\eta }\pi (\theta _{1},\theta _{2})\Big |_{\theta _{2}=0}. \end{aligned}$$

Now we prove that (13) holds at *k=m+1*. Let $\rho (\theta _{1},\theta _{2})= {}^{c}\mathcal {D}_{0,\theta _{2}}^{m\eta }\pi (\theta _{1},\theta _{2})$. By (14) and (16) we have

17 $$\begin{aligned} \begin{aligned}&\mathbb {Y}_{\theta _{2}\upsilon }\left[ {}^{c}\mathcal {D}_{0,\theta _{2}}^{k\eta }\rho (\theta _{1},\theta _{2})\right] =\mathbb {Y}_{\theta _{2}\upsilon }\left[ {}^{c}\mathcal {D}_{0,\theta _{2}}^{\eta }\rho (\theta _{1},\theta _{2})\right] = \upsilon ^{-\eta }\rho ^{\mathbb {Y}}_{\theta _{2}}(\theta _{1},\upsilon )-\upsilon ^{-\eta +1}\rho (\theta _{1},0)\\&= \upsilon ^{-\eta }\mathbb {Y}_{\theta _{2}\upsilon }{}^{c}\mathcal {D}_{0,\theta _{2}}^{m\eta }\pi (\theta _{1},\theta _{2})-\upsilon ^{-\eta +1}\,\,{}^{c}\mathcal {D}_{0,\theta _{2}}^{m\eta }\pi (\theta _{1},\theta _{2})\Big |_{\theta _{2}=0}\\&= \upsilon ^{-\eta }\left[ \upsilon ^{-m\eta }\pi ^{\mathbb {Y}}_{\theta _{2}}(\theta _{1},\upsilon )-\sum _{j=0}^{m-1}\upsilon ^{(j-m)\eta +1}\,{}^{c}\mathcal {D}_{0,\theta _{2}}^{j\eta }\pi (\theta _{1},\theta _{2})\Big |_{\theta _{2}=0}\right] \\ &-\upsilon ^{-\eta +1}\,\,{}^{c}\mathcal {D}_{0,\theta _{2}}^{m\eta }\pi (\theta _{1},\theta _{2})\Big |_{\theta _{2}=0}\\&=\upsilon ^{-(m+1)\eta }\pi ^{\mathbb {Y}}_{\theta _{2}}(\theta _{1},\upsilon )-\sum _{j=0}^{m-1}\upsilon ^{(j-(m+1)))\eta +1}\,{}^{c}\mathcal {D}_{0,\theta _{2}}^{j\eta }\pi (\theta _{1},\theta _{2})\Big |_{\theta _{2}=0}\\ &-\upsilon ^{\eta +1}\,\,{}^{c}\mathcal {D}_{0,\theta _{2}}^{\eta }\pi (\theta _{1},\theta _{2})\Big |_{\theta _{2}=0}\\&=\upsilon ^{-(m+1)\eta }\pi ^{\mathbb {Y}}_{\theta _{2}}(\theta _{1},\upsilon )-\sum _{j=0}^{m}\upsilon ^{j-(m+1)\eta +1}\,{}^{c}\mathcal {D}_{0,\theta _{2}}^{j\eta }\pi (\theta _{1},\theta _{2})\Big |_{\theta _{2}=0}\\&=\upsilon ^{-k\eta }\pi ^{\mathbb {Y}}_{\theta _{2}}(\theta _{1},\upsilon )-\sum _{j=0}^{k-1}\upsilon ^{(j-k)\eta +1}\,{}^{c}\mathcal {D}_{0,\theta _{2}}^{j\eta }\pi (\theta _{1},\theta _{2})\Big |_{\theta _{2}=0} \end{aligned} \end{aligned}$$

The proof is completed. *□*

By^46^, we have

18 $$\begin{aligned} \sum _{j=0}^{\infty }\pi _{j}(\theta _{1})(\theta _{2}-\theta _{20})^{j\eta }=\pi _{0}(\theta _{1})(\theta _{2}-\theta _{20})^{0}+\pi _{1}(\theta _{1})(\theta _{2}-\theta _{20})^{\eta }+\pi _{2}(\theta _{1})(\theta _{2}-\theta _{20})^{2\eta }+\cdots \end{aligned}$$

where $\theta _{1}= (\theta _{11},\theta _{12},\cdots ,\theta _{1n})\in \mathbb {R}^{n}, n\in \mathbb {N}$. To construct a residual power series solution in the Yang domain, we need the transformed function to admit a suitable expansion. The following lemma establishes the MFPS form of the Yang transform, which allows the RPSM coefficients to be determined explicitly.

### Lemma 2.5

Let *π* be an order-exponential map on $\mathbb {R}^{n}\times [0,\infty )$. The MFPS of $\pi ^{\mathbb {Y}}_{\theta _{2}}$ is presented by

19 $$\begin{aligned} \pi ^{\mathbb {Y}}_{\theta _{2}}(\theta _{1},\upsilon )=\sum _{j=0}^{\infty }\upsilon ^{j\eta +1}f_{j}(\theta _{1})\,\,\,\,\,\,\,\,\,\upsilon >0, \end{aligned}$$

where $$\theta _{1}= (\theta _{11},\theta _{12},\cdots ,\theta _{1n})\in \mathbb {R}^{n}, n\in \mathbb {N}$$.

### Proof

Use the Taylor series to get

20 $$\begin{aligned} \pi (\theta _{1},\theta _{2})=f_{0}(\theta _{1})+f_{1}(\theta _{1})\dfrac{\theta _{2}^{\eta }}{\Gamma (\eta +1)}+f_{2}(\theta _{1})\dfrac{\theta _{2}^{2\eta }}{\Gamma (2\eta +1)}+\cdots \end{aligned}$$

Take Yang transform for (19)

21 $$\begin{aligned} \begin{aligned}&\mathbb {Y}_{\theta _{2}\upsilon }\pi (\theta _{1},\theta _{2})= \mathbb {Y}_{\theta _{2}\upsilon }f_{0}(\theta _{1})+f_{1}(\theta _{1})\dfrac{ \mathbb {Y}_{\theta _{2}\upsilon }\theta _{2}^{\eta }}{\Gamma (\eta +1)}+f_{2}(\theta _{1})\dfrac{ \mathbb {Y}_{\theta _{2}\upsilon }\theta _{2}^{2\eta }}{\Gamma (2\eta +1)}+\cdots \\&= \upsilon f_{0}(\theta _{1})+f_{1}(\theta _{1})\dfrac{\upsilon ^{\eta +1} \Gamma (\eta +1)}{\Gamma (\eta +1)}+f_{2}(\theta _{1})\dfrac{\upsilon ^{2\eta +1} \Gamma (2\eta +1)}{\Gamma (2\eta +1)}+\cdots \\ &=\sum _{j=0}^{\infty }\upsilon ^{j\eta +1}f_{j}(\theta _{1}) \end{aligned} \end{aligned}$$

*□*

For the correctness of the MFPS representation, it is important to relate the Yang domain series to the function’s initial data. The next lemma verifies the limiting behavior of the Yang transform near zero, ensuring consistency with the initial condition.

### Lemma 2.6

Let *π* be an order-exponential map on $\mathbb {R}^{n}\times [0,\infty )$. Then $\lim _{\upsilon \rightarrow 0}\upsilon \pi ^{\mathbb {Y}}_{\theta _{2}}(\theta _{1},\upsilon )=\pi (\theta _{1},0)$ for all $\theta _{1}\in \mathbb {R}^{n}$.

### Proof

Put $\pi (\theta _{1},0)=f_{0}(\theta _{1})$ to obtain the desired. *□*

Using the previous lemmas, we now establish the full MFPS representation of the Yang transform. This theorem provides the explicit series form used as the backbone of the Yang RPSM and enables computation of successive coefficients.

### Theorem 2.7

Let *π* be an order-exponential map on $\mathbb {R}^{n}\times [0,\infty )$. Then

22 $$\begin{aligned} \pi ^{\mathbb {Y}}_{\theta _{2}}(\theta _{1},\upsilon )=\sum _{j=0}^{\infty }\upsilon ^{j\eta +1}f_{j}(\theta _{1})\,\,\,\,\,\,\,\,\,(0<\eta \le 1) \end{aligned}$$

for all $\theta _{1}\in \mathbb {R}^{n}$ and *υ >0*, where $f_{j}(\theta _{1})={}^{c}\mathcal {D}_{0,\theta _{2}}^{j\eta }\pi (\theta _{1},\theta _{2})\Big |_{\theta _{2}=0}$.

### Proof

The form of Taylor’s series is given by

23 $$\begin{aligned} f_{1}(\theta _{1}) =\upsilon ^{-\eta -1} \pi ^{\mathbb {Y}}_{\theta _{2}}(\theta _{1},\upsilon )-\upsilon ^{-\eta } f_{0}(\theta _{1})-\upsilon ^{\eta } f_{2}(\theta _{1})-\upsilon ^{2\eta } f_{3}(\theta _{1})-\cdots \end{aligned}$$

By taking the limit of (23) when $\upsilon \rightarrow 0$ to obtain

24 $$\begin{aligned} f_{1}(\theta _{1}) =\lim _{\upsilon \rightarrow 0}\left[ \upsilon ^{-\eta -1} \pi ^{\mathbb {Y}}_{\theta _{2}}(\theta _{1},\upsilon )-\upsilon ^{-\eta } f_{0}(\theta _{1})\right] . \end{aligned}$$

By Lemma 2.6 we have $f_{1}(\theta _{1}) =\partial ^{\eta }_{\theta _{2}}\pi (\theta _{1},0).$ Similar, the form of Taylor’s series of $f_{2}$ will be

25 $$\begin{aligned} f_{2}(\theta _{1}) =\upsilon ^{-2\eta -1} \pi ^{\mathbb {Y}}_{\theta _{2}}(\theta _{1},\upsilon )-\upsilon ^{-2\eta } f_{0}(\theta _{1})-\upsilon ^{-\eta } f_{1}(\theta _{1})-\upsilon ^{\eta } f_{3}(\theta _{1})-\cdots \end{aligned}$$

Take the limit of (25) when $\upsilon \rightarrow 0$ to obtain

26 $$\begin{aligned} f_{2}(\theta _{1}) =\lim _{\upsilon \rightarrow 0}\left[ \upsilon ^{-2\eta +1} \pi ^{\mathbb {Y}}_{\theta _{2}}(\theta _{1},\upsilon )-\upsilon ^{-2\eta } f_{0}(\theta _{1})-\upsilon ^{-\eta } f_{1}(\theta _{1})\right] . \end{aligned}$$

By Lemmas 2.4 and 2.6 we have $f_{2}(\theta _{1}) =\partial ^{2\eta }_{\theta _{2}}\pi (\theta _{1},0).$ By the continuity, we indicate that $f_{j}(\theta _{1}) ={}^{c}\mathcal {D}_{0,\theta _{2}}^{j\eta }\pi (\theta _{1},0).$ *□*

In theorem above we get

$$\begin{aligned} \mathbb {Y}_{\theta _{2}\upsilon }\left[ ^{c}\mathcal {D}_{0,\theta _{2}}^{\eta }\pi (\theta _{1},\theta _{2})\right] =\sum _{j=0}^{\infty }\upsilon ^{j\eta +1}\,\, ^{c}\mathcal {D}_{0,\theta _{2}}^{j\eta }\pi (\theta _{1},\theta _{2})\Big |_{\theta _{2}=0}\,\,\,\,\,\,\,\,\,(0<\eta \le 1) \end{aligned}$$

for all $\theta _{1}\in \mathbb {R}^{n}$, *υ >0* and the inverse Yang transform will be

$$\begin{aligned} \pi (\theta _{1},\theta _{2})=\sum _{j=0}^{\infty }\dfrac{ ^{c}\mathcal {D}_{0,\theta _{2}}^{j\eta }\pi (\theta _{1},\theta _{2})\Big |_{\theta _{2}=0}}{\Gamma (j\eta +1)}\,\,\,\theta _{2}^{j\eta }\,\,\,\,\,\,\,\,\,(0<\eta \le 1) \end{aligned}$$

for all $\theta _{1}\in \mathbb {R}^{n}$ and $\theta _{2}>0$.

To justify the convergence of the proposed Yang RPSM, we need to bound the residual error of the truncated MFPS series. The following theorem provides an estimate for the residual term, demonstrating that the series constructed through the Yang RPSM converges under suitable conditions.

### Theorem 2.8

Let *π* be an order-exponential map on $\mathbb {R}^{n}\times [0,\infty )$. If

$$\begin{aligned} \Big |\upsilon ^{r}\mathbb {Y}_{\theta _{2}\upsilon }\left[ ^{c}\mathcal {D}_{0,\theta _{2}}^{(n+1)\eta }\pi (\theta _{1},\theta _{2})\right] \Big |\le M \end{aligned}$$

for all $0<\upsilon \le q$ and $0<\eta \le 1$ then the residual form $\mathcal {R}es_{n}(\theta _{1},\upsilon )$ of MFPS has

$$\begin{aligned} \Arrowvert \mathcal {R}es_{n}(\theta _{1},\upsilon )\Arrowvert \le \dfrac{M}{\upsilon ^{-(n+1)\eta }}. \end{aligned}$$

### Proof

Take the form of Taylor series as

27 $$\begin{aligned} \mathcal {R}es_{n}(\theta _{1},\upsilon ) = \pi ^{\mathbb {Y}}_{\theta _{2}}(\theta _{1},\upsilon )+\sum _{j=0}^{n}\upsilon ^{j\eta +1}f_{j}(\theta _{1}). \end{aligned}$$

By Theorem 2.7 we have

28 $$\begin{aligned} \mathcal {R}es_{n}(\theta _{1},\upsilon ) = \pi ^{\mathbb {Y}}_{\theta _{2}}(\theta _{1},\upsilon )+\sum _{j=0}^{n}\upsilon ^{j\eta +1}\,\,{}^{c}\mathcal {D}_{0,\theta _{2}}^{j\eta }\pi (\theta _{1},0). \end{aligned}$$

Multiply (28) by $\upsilon ^{-(n+1)\eta }$ to get

29 $$\begin{aligned} \upsilon ^{-(n+1)\eta }\mathcal {R}es_{n}(\theta _{1},\upsilon ) =\upsilon ^{-(n+1)\eta }\pi ^{\mathbb {Y}}_{\theta _{2}}(\theta _{1},\upsilon )+\sum _{j=0}^{n}\upsilon ^{j-(n+1)\eta +1}{}^{c}\mathcal {D}_{0,\theta _{2}}^{j\eta }\pi (\theta _{1},0). \end{aligned}$$

By Lemma 2.4 we have

30 $$\begin{aligned} \upsilon ^{-(n+1)\eta }\mathcal {R}es_{n}(\theta _{1},\upsilon )= \mathbb {Y}_{\theta _{2}\upsilon }\left[ {}^{c}\mathcal {D}_{0,\theta _{2}}^{(n+1)\eta }\pi (\theta _{1},\theta _{2})\right] . \end{aligned}$$

Hence

$$\begin{aligned} \Big |\upsilon ^{-(n+1)\eta }\mathcal {R}es_{n}(\theta _{1},\upsilon ) \Big |=\Big | \mathbb {Y}_{\theta _{2}\upsilon }\left[ ^{c}\mathcal {D}_{0,\theta _{2}}^{(n+1)\eta }\pi (\theta _{1},\theta _{2})\right] \Big |. \end{aligned}$$

That is, $$|\mathcal {R}es_{n}(\theta _{1},\upsilon )|\le \dfrac{M}{\upsilon ^{-(n+1)\eta }}$$. *□*

## The Yang RPSM methodology

With the Yang transform rules and fractional series structure established, we now develop the Yang RPSM. This section explains how the residual maps are constructed and how the sequence of approximations converges to the solution. We outline the procedure of the Yang RPSM for handling fractional order partial differential equations. Let us take into account the following FPDE:

31 $$\begin{aligned} {}^{c}\mathcal {D}_{0,\theta _{2}}^{\eta }\pi (\theta _{1},\theta _{2})= \mathcal {N}(\pi (\theta _{1},\theta _{2}))+\mathcal {R}(\pi (\theta _{1},\theta _{2}))\,\,\,\,\,\,\,\,\,\,\,\,\,\,\,\,\,\,\,\,\,\, \eta \in (0,1] \end{aligned}$$

where $\mathcal {N}$ denotes nonlinear part depending on the map $\pi (\theta _{1},\theta _{2})$. The following initial conditions play important role role in determining the overall behaviour of terms $\mathcal {R}$ and $\mathcal {N}$.

32 $$\begin{aligned} \pi (\theta _{1},0)=h_{0}(\theta _{1}). \end{aligned}$$

Take the Yang transform $\mathbb {Y}_{\theta _{2}\upsilon }$ of Eq. (31)

33 $$\begin{aligned} \mathbb {Y}_{\theta _{2}\upsilon }\left[ {}^{c}\mathcal {D}_{0,\theta _{2}}^{\eta }\pi (\theta _{1},\theta _{2})\right] = \mathbb {Y}_{\theta _{2}\upsilon }\left[ \mathcal {N}(\pi (\theta _{1},\theta _{2}))\right] +\mathbb {Y}_{\theta _{2}\upsilon }\left[ \mathcal {R}(\pi (\theta _{1},\theta _{2}))\right] . \end{aligned}$$

By Lemma 2.2 and using Eq. (32), the Yang transform $\mathbb {Y}_{\theta _{2}\upsilon }$ for the left side of Eq. (33) becomes

34 $$\begin{aligned} \mathbb {Y}_{\theta _{2}\upsilon }\left[ {}^{c}\mathcal {D}_{0,\theta _{2}}^{\eta }\pi (\theta _{1},\theta _{2})\right] =\upsilon ^{-\eta }\pi ^{\mathbb {Y}}_{\theta _{2}}(\theta _{1},\upsilon )- \upsilon ^{-\eta -1}h_{0}(\theta _{1}). \end{aligned}$$

Hence from Eq. (33) and Eq. (34) we get that

35 $$\begin{aligned} \pi ^{\mathbb {Y}}_{\theta _{2}}(\theta _{1},\upsilon )=\upsilon h_{0}(\theta _{1})+\upsilon ^{\eta }\mathbb {N}_{\theta _{2}}( \theta _{1},\upsilon )+\upsilon ^{\eta }\mathbb {R}_{\theta _{2}} (\theta _{1},\upsilon ), \end{aligned}$$

where $\mathbb {N}_{\theta _{2}}( \theta _{1},\upsilon )=\mathbb {Y}_{\theta _{2}\upsilon }[\mathcal {N}(\pi (\theta _{1},\theta _{2}))]$ and $\mathbb {R}_{\theta _{2}} (\theta _{1},\upsilon )=\mathbb {Y}_{\theta _{2}\upsilon }[\mathcal {R}(\pi (\theta _{1},\theta _{2}))].$ Suppose that the following power series is a solution to Eq. (33) which can be represented through an expansion:

36 $$\begin{aligned} \pi ^{\mathbb {Y}}_{\theta _{2}}(\theta _{1},\upsilon )=\sum _{j=0}^{\infty }\upsilon ^{j\eta +1}h_{j}(\theta _{1}). \end{aligned}$$

By Lemma 2.2, we can write the partial sums sequence $\left<\pi ^{\mathbb {Y}n}_{\theta _{2}}\right>_{n\in \mathbb {N}\cup \{0\}}$ of the series above in Eq. (36) as

37 $$\begin{aligned} \pi ^{\mathbb {Y}n}_{\theta _{2}}(\theta _{1},\upsilon )=\upsilon h_{0}(\theta _{1})+\sum _{j=1}^{n}\upsilon ^{j\eta +1}h_{j}(\theta _{1}). \end{aligned}$$

Next, we structure the Yang residual function, $\mathbb {Y}_{\theta _{2}\upsilon }\mathcal {R}es\,\,\pi (\theta _{1},\upsilon )$ for Eq. (35) as follow:

38 $$\begin{aligned} \mathbb {Y}_{\theta _{2}\upsilon }\mathcal {R}es\,\,\pi (\theta _{1},\upsilon )=\pi ^{\mathbb {Y}}_{\theta _{2}}(\theta _{1},\upsilon )-\upsilon h_{0}(\theta _{1})-\upsilon ^{\eta }\mathbb {N}_{\theta _{2}} (\theta _{1},\upsilon )-\upsilon ^{\eta }\mathbb {R}_{\theta _{2}} (\theta _{1},\upsilon ) \end{aligned}$$

and the *n*-th Yang residual map $\mathbb {Y}_{\theta _{2}\upsilon }\mathcal {R}es_{n}\,\,\pi (\theta _{1},\upsilon )$ is

39 $$\begin{aligned} \mathbb {Y}_{\theta _{2}\upsilon }\mathcal {R}es_{n}\,\,\pi (\theta _{1},\upsilon )=\pi ^{\mathbb {Y}n}_{\theta _{2}}(\theta _{1},\upsilon )-\upsilon h_{0}(\theta _{1})-\upsilon ^{\eta }\mathbb {N}_{\theta _{2}}^{n} (\theta _{1},\upsilon )-\upsilon ^{\eta }\mathbb {R}_{\theta _{2}}^{n} (\theta _{1},\upsilon ). \end{aligned}$$

Note that $\mathbb {Y}_{\theta _{2}\upsilon }\mathcal {R}es\,\,\pi (\theta _{1},\upsilon )=0$ and $\lim _{n\rightarrow \infty }\mathbb {Y}_{\theta _{2}\upsilon }\mathcal {R}es_{n}\,\,\pi (\theta _{1},\upsilon )=\mathbb {Y}_{\theta _{2}\upsilon }\mathcal {R}es\,\,\pi (\theta _{1},\upsilon )$ for all *υ >0*. In general,

$$\begin{aligned} \lim _{\upsilon \rightarrow 0}\upsilon ^{-n\eta -1}\mathbb {Y}_{\theta _{2}\upsilon }\mathcal {R}es\,\,\pi (\theta _{1},\upsilon )= \lim _{\upsilon \rightarrow 0}\upsilon ^{-n\eta -1}\mathbb {Y}_{\theta _{2}\upsilon }\mathcal {R}es_{n}\,\,\pi (\theta _{1},\upsilon )=0 \end{aligned}$$

for $0<\eta \le 1$. To determine $h_{n}(\theta _{1})$, we will solve the following:

40 $$\begin{aligned} \lim _{\upsilon \rightarrow 0}\upsilon ^{-n\eta -1}\mathbb {Y}_{\theta _{2}\upsilon }\mathcal {R}es_{n}\,\,\pi (\theta _{1},\upsilon )=0 \end{aligned}$$

for all *n=1,2,3,⋯* and then we substitute the forms of the terms, $h_{n}(\theta _{1})$ into Eq. (37) to get the *n*-th solutions $\pi ^{\mathbb {Y}n}_{\theta _{2}}(\theta _{1},\upsilon )$ of Eq. (35). Finally, we take the inverse Yang transform of the *n*-th solutions $\pi ^{\mathbb {Y}n}_{\theta _{2}}(\theta _{1},\upsilon )$ to get the *n*-th solutions $\pi ^{\mathbb {Y}n}_{\theta _{2}}(\theta _{1},\theta _{2})$ of Eq. (31).

## Stability analysis of the Yang RPSM

Throughout this work, solutions of the MCFKE (3) are sought in appropriate fractional Sobolev spaces $H^{5}(\Omega )$. Specifically, we assume that $\pi (\cdot ,\theta _2) \in H^{5}(\Omega ) \quad \text {for a.e. } \theta _2 \ge 0,$ so that the third- and fifth-order spatial derivatives are well defined, and $\pi (\theta _1,\cdot ) \in AC([0,T])$ with respect to the temporal variable, ensuring the existence of the Caputo fractional derivative of order $0<\eta \le 1$. For the purposes of series construction and residual analysis, we further assume that *π* is sufficiently smooth, namely $\pi \in C^{5,1}(\Omega \times [0,T])$. These regularity assumptions are standard in the analysis of high order fractional partial differential equations and provide the functional setting required for the application of the Yang RPSM. In this section, we establish the stability of the Yang RPSM when applied to the MCFKE (3). The analysis is carried out in the Yang-transform domain and focuses on three key ingredients: stability of the recursive construction of the coefficients, stability under perturbations in the initial data, and decay and boundedness of the Yang residual. This guarantees that the method produces stable and convergent approximate analytical solutions. Although the convergence of the Yang RPSM is guaranteed by Theorem (2.8), the practical rate of convergence and the number of terms required for accurate approximation depend strongly on the dispersive parameters *λ* and *λ '*. The third-order dispersion coefficient *λ* and, more significantly, the fifth-order dispersion coefficient *λ '* control the strength of high-frequency wave components in the MCFKE. As the magnitudes of *λ* and *λ '* increase, higher spatial derivatives contribute more strongly to the recursive coefficients of the MFPS expansion. This leads to faster growth of higher-order terms in the series and effectively reduces the convergence radius with respect to the temporal variable $\theta _2$. Consequently, a larger number of Yang RPSM terms is required to maintain a prescribed accuracy when strong dispersion is present. In contrast, the Yang RPSM converges with fewer terms. This behavior is consistent with the theoretical residual bound in Theorem (2.8), where the residual magnitude depends on higher-order fractional derivatives that are amplified by strong dispersive operators. Let the *n*-term Yang domain approximation be

$$\pi ^{\mathbb {Y}}_{n}(\theta _{1},\upsilon ) = \upsilon h_{0}(\theta _{1}) + \sum _{j=1}^{n} \upsilon ^{j\eta +1} h_{j}(\theta _{1}),$$

and define the associated Yang residual map

$$\mathcal {R}es_{n}(\theta _{1},\upsilon ) = \pi ^{\mathbb {Y}}_{n}(\theta _{1},\upsilon ) - \upsilon h_{0}(\theta _{1}) - \upsilon ^{\eta } \mathcal {N}_{n}(\theta _{1},\upsilon ) - \upsilon ^{\eta } \mathcal {R}_{n}(\theta _{1},\upsilon ),$$

where $\mathcal {N}_{n}$ and $\mathcal {R}_{n}$ represent the truncated nonlinear and dispersive contributions as defined earlier. The Yang RPSM determines $h_{n}(\theta _{1})$ by enforcing the condition

$$\begin{aligned} \lim _{\upsilon \rightarrow 0}\upsilon ^{-n\eta -1} \mathcal {R}es_{n}(\theta _{1},\upsilon )=0. \end{aligned}$$

To ensure that the Yang RPSM produces a stable sequence of approximations, we first examine the behavior of its recursive coefficient construction. The next theorem confirms that this recursion is well controlled and does not amplify errors. Before stating the stability result, we specify the functional setting of the contractive argument. Let $\mathcal {B}$ denote the Banach space of Yang-transformed functions $\pi ^{\mathbb {Y}}(\theta _1,\upsilon )$ that are continuous for $\upsilon \in (0,\upsilon _0]$ and bounded as $\upsilon \rightarrow 0$, equipped with the supremum norm

$$\Vert \pi ^{\mathbb {Y}}\Vert _{\mathcal {B}} = \sup _{0<\upsilon \le v_0} |\pi ^{\mathbb {Y}}(\theta _1,\upsilon )|.$$

The recursive coefficients generated by the Yang RPSM define a mapping on $\mathcal {B}$, and stability is established with respect to this norm.

### Definition 4.1

Let *B* be the Banach space of Yang-transformed functions equipped with the norm $\Vert \cdot \Vert$. The nonlinear operator *N(· )* and dispersive operator *R(· )* are said to satisfy the Lipschitz condition if there exists a constant *L > 0* such that for all $\pi _1, \pi _2 \in B$,

$$\Vert N(\pi _1) - N(\pi _2)\Vert \le L \Vert \pi _1 - \pi _2\Vert , \quad \Vert R(\pi _1) - R(\pi _2)\Vert \le L \Vert \pi _1 - \pi _2\Vert .$$

The constant *L* is called the Lipschitz constant and is used to establish the contraction and stability of the Yang-RPSM recursive formulation.

### Theorem 4.2

Assume the nonlinear operator $\mathcal {N}(\cdot )$ and dispersive operator $\mathcal {R}(\cdot )$ appearing in the MCFKE (3) are locally Lipschitz in the spatial Sobolev space $H^{5}(\Omega )$. Then the recursive map

$$\begin{aligned} h_{n}(\theta _{1}) \longmapsto h_{n+1}(\theta _{1}) \end{aligned}$$

defined by the Yang RPSM is a contraction in the Yang-transform domain for all $0 < \eta \le 1$, and hence the sequence $\{h_{n}\}$ is stable.

### Proof

From the definition of the Yang residual,

$$\mathcal {R}es_{n}(\theta _{1},\upsilon ) = \upsilon ^{n\eta +1} h_{n}(\theta _{1}) + \mathcal {O}(\upsilon ^{(n+1)\eta +1}),$$

and the stability condition requires

$$\begin{aligned} \lim _{\upsilon \rightarrow 0} \upsilon ^{-n\eta -1} \mathcal {R}es_{n}(\theta _{1},\upsilon )=0, \end{aligned}$$

which gives a unique solvability condition for $h_{n}(\theta _{1})$. Since $\mathcal {N}$ and $\mathcal {R}$ are Lipschitz, we have

$$\Vert \mathcal {N}(\pi _{n})-\mathcal {N}(\pi _{n-1})\Vert \le L \Vert \pi _{n} - \pi _{n-1}\Vert ,$$

and similarly for $\mathcal {R}(\cdot )$. Because the Yang kernel $\upsilon ^{\eta }$ satisfies $0<\upsilon ^\eta <1$ for $\upsilon \in (0,1)$, the recursive update for $h_{n}$ carries a multiplicative factor strictly less than one, ensuring contractivity:

$$\begin{aligned} \Vert h_{n+1}-h_{n}\Vert \le \upsilon ^\eta L \Vert h_{n}-h_{n-1}\Vert . \end{aligned}$$

Thus the recursion is contractive, proving stability. *□*

After establishing stability of the recursion, we next verify that the method responds smoothly to changes in the initial data. The following theorem shows that the Yang RPSM is Lipschitz stable with respect to such perturbations.

### Theorem 4.3

Let the initial condition be perturbed as

$$\begin{aligned} \tilde{h}_{0}(\theta _{1})=h_{0}(\theta _{1})+\varepsilon (\theta _{1}). \end{aligned}$$

Then the Yang RPSM approximation satisfies

$$\Vert \tilde{\pi }(\theta _{1},\theta _{2})-\pi (\theta _{1},\theta _{2})\Vert \le C\,\theta _{2}^{\eta } \Vert \varepsilon (\theta _{1})\Vert ,$$

for some constant *C>0*. Hence the solution is Lipschitz stable.

### Proof

By linearity of the Yang transform,

$$\tilde{\pi }^{\mathbb {Y}}(\theta _{1},\upsilon )-\pi ^{\mathbb {Y}}(\theta _{1},\upsilon ) = \upsilon \varepsilon (\theta _{1}).$$

Applying the inverse Yang transform yields

$$\tilde{\pi }(\theta _{1},\theta _{2})-\pi (\theta _{1},\theta _{2}) = \frac{\theta _{2}^\eta }{\Gamma (\eta +1)} \varepsilon (\theta _{1}).$$

Taking norms gives the desired bound with

$$\begin{aligned} C = \frac{1}{\Gamma (\eta +1)}. \end{aligned}$$

Thus, the solution depends continuously on the initial data, proving Lipschitz stability. *□*

Suppose the condition in Theorem(2.8) holds, namely,

$$\left| \upsilon ^{-(n+1)\eta } \mathbb {Y}_{\theta _{2}\upsilon }\left[ ^{c}\mathcal {D}^{(n+1)\eta }_{0,\theta _{2}} \pi (\theta _{1},\theta _{2})\right] \right| \le M.$$

Then the residual satisfies

$$\begin{aligned} \Vert \mathcal {R}es_{n}(\theta _{1},\upsilon )\Vert \le M \upsilon ^{(n+1)\eta }, \end{aligned}$$

and the Yang RPSM series converges for all $0<\eta \le 1$. Because Theorem(2.8) directly gives the bound

$$\upsilon ^{-(n+1)\eta } \mathcal {R}es_{n}(\theta _{1},\upsilon ) = \mathbb {Y}_{\theta _{2}\upsilon }\!\left[ ^{c}\mathcal {D}^{(n+1)\eta }_{0,\theta _{2}} \pi \right] ,$$

whose magnitude is uniformly bounded by *M*. Multiplying both sides by $\upsilon ^{(n+1)\eta }$ yields

$$\begin{aligned} |\mathcal {R}es_{n}(\theta _{1},\upsilon )| \le M\upsilon ^{(n+1)\eta }, \end{aligned}$$

which converges to zero as $n\rightarrow \infty$ since $\upsilon ^\eta <1$ for $\upsilon \in (0,1)$. Hence the Yang RPSM series converges, and residual errors do not accumulate, establishing residual stability. Therefore, the Yang RPSM is a stable and reliable analytical numerical method for solving the MCFKE. The analysis confirms that the Yang RPSM is stable and convergent. Its recursive structure is well controlled, it depends continuously on initial data, and its residual error decreases with each iteration. Thus, the method provides reliable approximations for the MCFKE (3).

## Some applications

As recalled, the fractional Kawahara equation extends the classical Kawahara equation, a higher-order dispersive PDE, by incorporating fractional derivatives in space or time. This generalization makes it particularly useful for numerical simulations of chemical signaling processes, where standard diffusion or wave equations fail to capture memory effects, anomalous transport, or nonlocal interactions. Its applications include modeling anomalous diffusion in signaling pathways, where chemical signaling inside cells often exhibits subdiffusion or superdiffusion due to molecular crowding, heterogeneous cytoplasmic structure, or unbinding kinetics. The fractional Kawahara equation can more accurately simulate these anomalous transport dynamics than classical models.

**Fig. 1:**
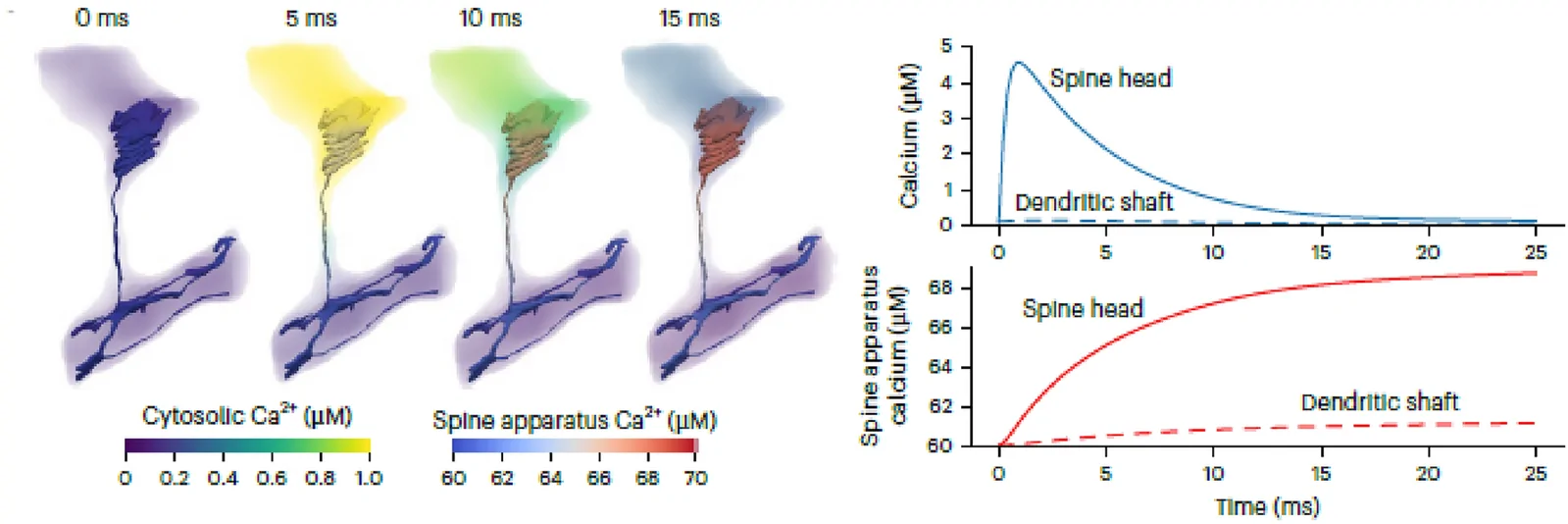
Simulation of Ca${}^{2+}$ dynamics in a dendritic spine.

**Fig. 2:**
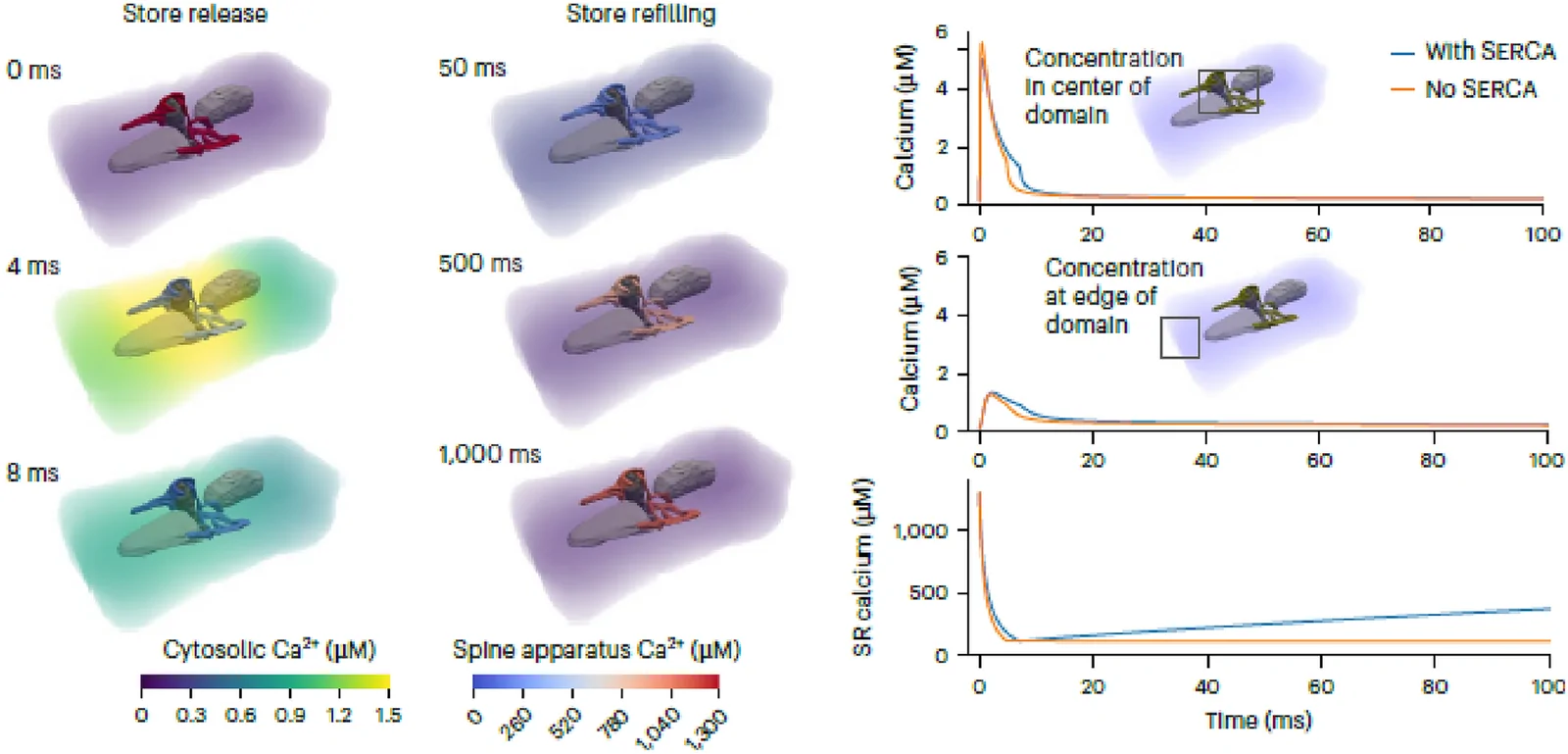
Simulation of Ca${}^{2+}$ dynamics in a Cardiomyocyte.

In signal propagation across dendritic spines and neural tissue, fractional Kawahara type models describe how nonlinear dispersive waves of signals propagate with attenuation and distortion along dendritic structures, closely mimicking real neuronal signaling. For example, fractional Kawahara type models can describe the simulation of Ca${}^{2+}$ dynamics in a dendritic spine, as shown in Fig. (1)^47^, and in a cardiomyocyte, as shown in Fig. 2^47^. This section employs the Yang RPSM to get some solution of MCFKE (3) in two cases: firstly, with approximate initial formula. The fractional Kawahara framework provides a robust foundation for simulating biochemical signaling processes, particularly calcium (Ca$^{2+}$) dynamics, where classical diffusion or wave equations fail to capture memory effects, anomalous transport, and higher order dispersive behaviors. In the MCFKE (3), the inclusion of a Caputo derivative of order $0 < \eta \le 1$ plays a central biological role. When *η < 1*, the fractional operator introduces a long tailed temporal memory kernel that models the history dependent behavior of Ca$^{2+}$ in crowded cytoplasm. Because Ca$^{2+}$ ions bind to buffers, receptors, channels, and organelles, the concentration at a given time depends on a broad range of earlier states. This generates a non–Markovian, viscoelastic temporal response that fractional calculus captures in a natural and mathematically rigorous way. Furthermore, intracellular Ca$^{2+}$ spreads subdiffusively, its mean–square displacement grows like $t^\eta$ with *0< η < 1*, due to molecular crowding, cytoskeletal obstacles, and heterogeneous organelle distributions. Fractional dynamics directly encode this anomalous diffusion behavior, producing attenuated wavefronts and slowed propagation that match experimentally observed Ca$^{2+}$ behavior. Thus, the term ${}^{c}\mathcal {D}^{\eta }_{0,\theta _2}\pi (\theta _1,\theta _2)$ models both biochemical memory and anomalous transport, making the MCFKE (3) biologically realistic for intracellular signaling environments. Figures 1 and 2 illustrate the biological relevance of the MCFKE (3). Figure 1 shows Ca$^{2+}$ dynamics within a dendritic spine, a geometrically constrained neuronal microdomain where molecular crowding and spine-neck bottlenecks produce strong subdiffusive behavior and long range memory. The MCFKE (3), evaluated through the Yang RPSM, reproduces localized Ca$^{2+}$ elevation in the spine head, attenuated propagation through the narrow neck, and the small oscillations and dispersive spreading observed in experimental Ca$^{2+}$ imaging. The fractional term models buffer mediated temporal memory, the nonlinear term captures amplification, and the dispersive terms shape the waveform as it moves from head to neck. Similarly, Fig. 2 depicts Ca$^{2+}$ propagation in a cardiomyocyte, a larger and more structurally organized domain where Ca$^{2+}$ waves exhibit strong nonlinear amplification followed by rapid attenuation. The high order dispersion term *λ ' < 0* plays a crucial role here, mimicking the suppression of short wavelength components caused by the sarcoplasmic reticulum network, mitochondrial barriers, and the high buffering capacity of cardiac cytoplasm. In both figures, the numerical waveforms shown are produced using approximate or exact Yang RPSM solutions of the MCFKE (3), demonstrating that the model and method accurately reproduce physiologically constrained Ca$^{2+}$ signaling behavior. In summary, the fractional order *η*, the nonlinear advection, and the dispersive parameters $(\lambda ,\lambda ')$ in the MCFKE (3) are not merely mathematical constructs but correspond directly to experimentally quantifiable biophysical properties: cytoplasmic viscosity, buffer mediated memory, organelle geometry, damping mechanisms, and nonlinear Ca$^{2+}$ release. The model thus provides a physiologically grounded, multiscale mathematical description of Ca$^{2+}$ wave dynamics in both neuronal and cardiac environments, as demonstrated in Figs. 1 and 2.

Consider the following MCFKE

41 $$\begin{aligned} \left\{ \begin{aligned}&{}^{c}\mathcal {D}_{0,\theta _{2}}^{\eta }\pi (\theta _{1},\theta _{2})+\pi ^{\mathbb {Y}2}(\theta _{1},\theta _{2})\frac{\partial \pi (\theta _{1},\theta _{2})}{\partial \theta _{1}}+\lambda \frac{\partial ^{3} \pi (\theta _{1},\theta _{2})}{\partial \theta _{1}^{3}}+\lambda '\frac{\partial ^{5} \pi (\theta _{1},\theta _{2})}{\partial \theta _{1}^{5}}=0,\\&\pi (\theta _{1},0)=0.9487\frac{\lambda }{\sqrt{-\lambda '}}+0.0474\frac{\lambda ^{2}}{\lambda '\sqrt{-\lambda '}} \theta _{1}^{2}+0.0021\frac{\lambda ^{3}}{\lambda '^{2}\sqrt{-\lambda '}} \theta _{1}^{4}+0.3584\frac{\lambda ^{4}}{\lambda '^{3}\sqrt{-\lambda '}} \theta _{1}^{6} \end{aligned} \right. \end{aligned}$$

for $0<\eta \le 1$. The polynomial initial condition in MCFKE (41) is used because it provides a smooth, localized starting profile consistent with the gradual onset of biochemical signals. Its smoothness avoids nonphysical singularities in fractional derivatives and reflects realistic early-time Ca$^{2+}$ activation. Take Yang transforms of (41)

42 $$\begin{aligned} \begin{aligned}&\pi ^{\mathbb {Y}}_{\theta _{2}}(\theta _{1},\upsilon )=\frac{\upsilon }{\sqrt{-\lambda '}}[0.9487\lambda +0.0474\frac{\lambda ^{2}}{\lambda '} \theta _{1}^{2}+0.0021\frac{\lambda ^{3}}{\lambda '^{2}} \theta _{1}^{4}+0.3584\frac{\lambda ^{4}}{\lambda '^{3}} \theta _{1}^{6}]\\&-\upsilon ^{\eta }\mathbb {Y}_{\theta _{2}}\left[ \Big [\mathbb {Y}_{\theta _{2}}^{-1}(\pi ^{\mathbb {Y}}_{\theta _{2}}(\theta _{1},\upsilon ))\Big ]^{2}\frac{\partial }{\partial \theta _{1}}[\mathbb {Y}_{\theta _{2}}^{-1}(\pi ^{\mathbb {Y}}_{\theta _{2}}(\theta _{1},\upsilon ))]\right] \\&-\lambda \upsilon ^{\eta }\mathbb {Y}_{\theta _{2}}\left[ \frac{\partial ^{3}}{\partial \theta _{1}^{3}}[\mathbb {Y}_{\theta _{2}}^{-1}(\pi ^{\mathbb {Y}}_{\theta _{2}}(\theta _{1},\upsilon ))]\right] -\upsilon ^{\eta }\lambda '\mathbb {Y}_{\theta _{2}}\left[ \frac{\partial ^{5}}{\partial \theta _{1}^{5}}[\mathbb {Y}_{\theta _{2}}^{-1}(\pi ^{\mathbb {Y}}_{\theta _{2}}(\theta _{1},\upsilon ))] \right] . \end{aligned} \end{aligned}$$

The sequence $\left<\pi ^{\mathbb {Y}}_{ \theta _{2} n}\right>_{n\in \mathbb {N}\cup \{0\}}$ given by

43 $$\begin{aligned} \pi ^{\mathbb {Y}}_{ \theta _{2} n}(\theta _{1},\upsilon )=\frac{\upsilon }{\sqrt{-\lambda '}}[0.9487\lambda +0.0474\frac{\lambda ^{2}}{\lambda '} \theta _{1}^{2}+0.0021\frac{\lambda ^{3}}{\lambda '^{2}} \theta _{1}^{4}+0.3584\frac{\lambda ^{4}}{\lambda '^{3}} \theta _{1}^{6}]+\sum _{j=1}^{n}\upsilon ^{j\eta +1}g_{j}(\theta _{1}). \end{aligned}$$

The *n*-th structure for (42) is

44 $$\begin{aligned} \begin{aligned}&\pi ^{\mathbb {Y}}_{ \theta _{2} n}(\theta _{1},\upsilon )=\frac{\upsilon }{\sqrt{-\lambda '}}[0.9487\lambda +0.0474\frac{\lambda ^{2}}{\lambda '} \theta _{1}^{2}+0.0021\frac{\lambda ^{3}}{\lambda '^{2}} \theta _{1}^{4}+0.3584\frac{\lambda ^{4}}{\lambda '^{3}} \theta _{1}^{6}]\\&-\upsilon ^{\eta }\mathbb {Y}_{\theta _{2}}\left[ [\mathbb {Y}_{\theta _{2}}^{-1}(\pi ^{\mathbb {Y}}_{ \theta _{2} n}(\theta _{1},\upsilon ))]^{2}\frac{\partial }{\partial \theta _{1}}[\mathbb {Y}_{\theta _{2}}^{-1}(\pi ^{\mathbb {Y}}_{ \theta _{2} n}(\theta _{1},\upsilon ))]\right] \\&-\lambda \upsilon ^{\eta }\mathbb {Y}_{\theta _{2}}\left[ \frac{\partial ^{3}}{\partial \theta _{1}^{3}}[\mathbb {Y}_{\theta _{2}}^{-1}(\pi ^{\mathbb {Y}}_{ \theta _{2} n}(\theta _{1},\upsilon ))]\right] -\upsilon ^{\eta }\lambda '\mathbb {Y}_{\theta _{2}}\left[ \frac{\partial ^{5}}{\partial \theta _{1}^{5}}[\mathbb {Y}_{\theta _{2}}^{-1}(\pi ^{\mathbb {Y}}_{ \theta _{2} n}(\theta _{1},\upsilon ))] \right] . \end{aligned} \end{aligned}$$

The map $\mathbb {Y}_{\theta _{2}}\mathcal {R}es\,\,\pi (\theta _{1},\upsilon )$ for (42) is given by

45 $$\begin{aligned} \begin{aligned}&\mathbb {Y}_{\theta _{2}}\mathcal {R}es\,\,\pi (\theta _{1},\upsilon )= \pi ^{\mathbb {Y}}_{\theta _{2}}(\theta _{1},\upsilon )-\frac{\upsilon }{\sqrt{-\lambda '}}[0.9487\lambda +0.0474\frac{\lambda ^{2}}{\lambda '} \theta _{1}^{2}+0.0021\frac{\lambda ^{3}}{\lambda '^{2}} \theta _{1}^{4}\\ &+0.3584\frac{\lambda ^{4}}{\lambda '^{3}} \theta _{1}^{6}]+\upsilon ^{\eta }\mathbb {Y}_{\theta _{2}}\left[ \Big [\mathbb {Y}_{\theta _{2}}^{-1}(\pi ^{\mathbb {Y}}_{\theta _{2}}(\theta _{1},\upsilon ))\Big ]^{2}\frac{\partial }{\partial \theta _{1}}[\mathbb {Y}_{\theta _{2}}^{-1}(\pi ^{\mathbb {Y}}_{\theta _{2}}(\theta _{1},\upsilon ))]\right] \\&+\lambda \upsilon ^{\eta }\mathbb {Y}_{\theta _{2}}\left[ \frac{\partial ^{3}}{\partial \theta _{1}^{3}}[\mathbb {Y}_{\theta _{2}}^{-1}(\pi ^{\mathbb {Y}}_{\theta _{2}}(\theta _{1},\upsilon ))]\right] +\upsilon ^{\eta }\lambda '\mathbb {Y}_{\theta _{2}}\left[ \frac{\partial ^{5}}{\partial \theta _{1}^{5}}[\mathbb {Y}_{\theta _{2}}^{-1}(\pi ^{\mathbb {Y}}_{\theta _{2}}(\theta _{1},\upsilon ))] \right] \end{aligned} \end{aligned}$$

**Table 1:** Comparing AE for solving (41) at *η =1*, *λ =0.001*, *λ '=-1*, $\theta _{1} = 10$.

$\theta _{2}$	AE(Yang RPSM)	AE(NTDT)^40^	AE(RPSM)^42^
0.1	$2.30201E^{-13}$	$1.41553E^{-15}$	$1.41553E^{-15}$
0.2	$3.85041E^{-15}$	$4.68063E^{-14}$	$4.68063E^{-14}$
0.3	$0.60951E^{-14}$	$3.63910E^{-13}$	$3.63910E^{-13}$
0.4	$2.07857E^{-15}$	$1.56886E^{-12}$	$1.56886E^{-12}$
0.5	$1.21689E^{-14}$	$4.89617E^{-12}$	$4.89617E^{-12}$
0.6	$3.13576E^{-12}$	$1.24542E^{-11}$	$1.24542E^{-11}$
0.7	$6.36014E^{-13}$	$2.75069E^{-11}$	$2.75069E^{-11}$
0.8	$5.55128E^{-18}$	$5.47829E^{-11}$	$5.47829E^{-11}$
0.9	$3.10893E^{-12}$	$1.00810E^{-10}$	$1.00810E^{-10}$
1.0	$0.39014E^{-11}$	$1.74280E^{-10}$	$1.74280E^{-10}$

**Table 2:** Yang RPSM solutions of (41) at *λ =0.001*, *λ '=-1*, *η =0.25* and *η =0.5*.

			*η =0.25*			*η =0.50*	
$\theta _{2}$	$\theta _{1}$	Yang RPSM	NTDT^40^	HAT^36^	Yang RPSM	NTDT^40^	HAT^36^
20	0.2	$9.2911E^{-4}$	$9.2996E^{-4}$	$9.299E^{-4}$	$9.2992E^{-4}$	$9.2996E^{-4}$	$9.299E^{-4}$
	0.4	$9.2912E^{-4}$	$9.2996E^{-4}$	$9.299E^{-4}$	$9.2992E^{-4}$	$9.2996E^{-4}$	$9.299E^{-4}$
	0.6	$9.2909E^{-4}$	$9.2996E^{-4}$	$9.299E^{-4}$	$9.2992E^{-4}$	$9.2996E^{-4}$	$9.299E^{-4}$
	0.8	$9.2991E^{-4}$	$9.2996E^{-4}$	$9.299E^{-4}$	$9.2992E^{-4}$	$9.2996E^{-4}$	$9.299E^{-4}$
	1.0	$9.2988E^{-4}$	$9.2996E^{-4}$	$9.299E^{-4}$	$9.2992E^{-4}$	$9.2996E^{-4}$	$9.299E^{-4}$
10	0.2	$9.4389E^{-4}$	$9.4396E^{-4}$	$9.439E^{-4}$	$9.4391E^{-4}$	$9.4396E^{-4}$	$9.439E^{-4}$
	0.4	$9.4390E^{-4}$	$9.4396E^{-4}$	$9.439E^{-4}$	$9.4391E^{-4}$	$9.4396E^{-4}$	$9.439E^{-4}$
	0.6	$9.4391E^{-4}$	$9.4396E^{-4}$	$9.439E^{-4}$	$9.4391E^{-4}$	$9.4396E^{-4}$	$9.439E^{-4}$
	0.8	$9.4390E^{-4}$	$9.4396E^{-4}$	$9.439E^{-4}$	$9.4391E^{-4}$	$9.4396E^{-4}$	$9.439E^{-4}$
	1.0	$9.4384E^{-4}$	$9.4396E^{-4}$	$9.439E^{-4}$	$9.4391E^{-4}$	$9.4396E^{-4}$	$9.439E^{-4}$
0	0.2	$9.4859E^{-4}$	$9.4868E^{-4}$	$9.486E^{-4}$	$9.4862E^{-4}$	$9.4868E^{-4}$	$9.486E^{-4}$
	0.4	$9.4863E^{-4}$	$9.4868E^{-4}$	$9.486E^{-4}$	$9.4862E^{-4}$	$9.4868E^{-4}$	$9.486E^{-4}$
	0.6	$9.4867E^{-4}$	$9.4868E^{-4}$	$9.486E^{-4}$	$9.4862E^{-4}$	$9.4868E^{-4}$	$9.486E^{-4}$
	0.8	$9.4860E^{-4}$	$9.4868E^{-4}$	$9.486E^{-4}$	$9.4862E^{-4}$	$9.4868E^{-4}$	$9.486E^{-4}$
	1.0	$9.4861E^{-4}$	$9.4868E^{-4}$	$9.486E^{-4}$	$9.4862E^{-4}$	$9.4868E^{-4}$	$9.486E^{-4}$
*-10*	0.2	$9.4392E^{-4}$	$9.4396E^{-4}$	$9.439E^{-4}$	$9.4391E^{-4}$	$9.4396E^{-4}$	$9.439E^{-4}$
	0.4	$9.4390E^{-4}$	$9.4396E^{-4}$	$9.439E^{-4}$	$9.4391E^{-4}$	$9.4396E^{-4}$	$9.439E^{-4}$
	0.6	$9.4860E^{-4}$	$9.4868E^{-4}$	$9.486E^{-4}$	$9.4862E^{-4}$	$9.4868E^{-4}$	$9.486E^{-4}$
	0.8	$9.4391E^{-4}$	$9.4396E^{-4}$	$9.439E^{-4}$	$9.4391E^{-4}$	$9.4396E^{-4}$	$9.439E^{-4}$
	1.0	$9.4391E^{-4}$	$9.4396E^{-4}$	$9.439E^{-4}$	$9.4391E^{-4}$	$9.4396E^{-4}$	$9.439E^{-4}$
*-20*	0.2	$9.2992E^{-4}$	$9.2996E^{-4}$	$9.299E^{-4}$	$9.2991E^{-4}$	$9.2996E^{-4}$	$9.299E^{-4}$
	0.4	$9.2989E^{-4}$	$9.2996E^{-4}$	$9.299E^{-4}$	$9.2991E^{-4}$	$9.2996E^{-4}$	$9.299E^{-4}$
	0.6	$9.2992E^{-4}$	$9.2996E^{-4}$	$9.299E^{-4}$	$9.2991E^{-4}$	$9.2996E^{-4}$	$9.299E^{-4}$
	0.8	$9.2998E^{-4}$	$9.2996E^{-4}$	$9.299E^{-4}$	$9.2991E^{-4}$	$9.2996E^{-4}$	$9.299E^{-4}$
	1.0	$9.2991E^{-4}$	$9.2996E^{-4}$	$9.299E^{-4}$	$9.2991E^{-4}$	$9.2996E^{-4}$	$9.299E^{-4}$

with the *n*-th structure

46 $$\begin{aligned} \begin{aligned}&\mathbb {Y}_{\theta _{2}}\mathcal {R}es_{n}\,\,\pi (\theta _{1},\upsilon )= \pi ^{\mathbb {Y}}_{ \theta _{2} n}(\theta _{1},\upsilon )-\frac{\upsilon }{\sqrt{-\lambda '}}[0.9487\lambda +0.0474\frac{\lambda ^{2}}{\lambda '} \theta _{1}^{2}+0.0021\frac{\lambda ^{3}}{\lambda '^{2}} \theta _{1}^{4}\\ &+0.3584\frac{\lambda ^{4}}{\lambda '^{3}} \theta _{1}^{6}]+\upsilon ^{\eta }\mathbb {Y}_{\theta _{2}}\left[ [\mathbb {Y}_{\theta _{2}}^{-1}(\pi ^{\mathbb {Y}}_{ \theta _{2} n}(\theta _{1},\upsilon ))]^{2}\frac{\partial }{\partial \theta _{1}}[\mathbb {Y}_{\theta _{2}}^{-1}(\pi ^{\mathbb {Y}}_{ \theta _{2} n}(\theta _{1},\upsilon ))]\right] \\&+\lambda \upsilon ^{\eta }\mathbb {Y}_{\theta _{2}}\left[ \frac{\partial ^{3}}{\partial \theta _{1}^{3}}[\mathbb {Y}_{\theta _{2}}^{-1}(\pi ^{\mathbb {Y}}_{ \theta _{2} n}(\theta _{1},\upsilon ))]\right] +\upsilon ^{\eta }\lambda '\mathbb {Y}_{\theta _{2}}\left[ \frac{\partial ^{5}}{\partial \theta _{1}^{5}}[\mathbb {Y}_{\theta _{2}}^{-1}(\pi ^{\mathbb {Y}}_{ \theta _{2} n}(\theta _{1},\upsilon ))] \right] . \end{aligned} \end{aligned}$$

We calculate the terms of $\left<g_{n}\right>_{n\in \mathbb {N}\cup \{0\}}$ by

47 $$\begin{aligned} \lim _{\upsilon \rightarrow 0}\upsilon ^{-n\eta -1}\mathbb {Y}_{\theta _{2}}\mathcal {R}es_{n}\,\,\pi (\theta _{1},\upsilon )=0\,\,\,\,\,\,\,\, n=1,2,3,\cdots \end{aligned}$$

At *n=1*, by (43)

$$\begin{aligned} \pi ^{\mathbb {Y}}_{\theta _{2}1}(\theta _{1},\upsilon )=\frac{\upsilon }{\sqrt{-\lambda '}}[0.9487\lambda +0.0474\frac{\lambda ^{2}}{\lambda '} \theta _{1}^{2}+0.0021\frac{\lambda ^{3}}{\lambda '^{2}} \theta _{1}^{4}+0.3584\frac{\lambda ^{4}}{\lambda '^{3}} \theta _{1}^{6}]+\upsilon ^{\eta +1}g_{1}(\theta _{1}) \end{aligned}$$

and (46) will be

$$\begin{aligned} \begin{aligned}&\mathbb {Y}_{\theta _{2}}\mathcal {R}es_{1}\,\,\pi (\theta _{1},\upsilon )= \upsilon ^{\eta +1}g_{1}(\theta _{1}) +\upsilon ^{\eta }\mathbb {Y}_{\theta _{2}}\left[ [\mathbb {Y}_{\theta _{2}}^{-1}(\pi ^{\mathbb {Y}}_{\theta _{2}1}(\theta _{1},\upsilon ))]^{2}\frac{\partial }{\partial \theta _{1}}[\mathbb {Y}_{\theta _{2}}^{-1}(\pi ^{\mathbb {Y}}_{\theta _{2}1}(\theta _{1},\upsilon ))]\right] \\&+\lambda \upsilon ^{\eta }\mathbb {Y}_{\theta _{2}}\left[ \frac{\partial ^{3}}{\partial \theta _{1}^{3}}[\mathbb {Y}_{\theta _{2}}^{-1}(\pi ^{\mathbb {Y}}_{\theta _{2}1}(\theta _{1},\upsilon ))]\right] +\upsilon ^{\eta }\lambda '\mathbb {Y}_{\theta _{2}}\left[ \frac{\partial ^{5}}{\partial \theta _{1}^{5}}[\mathbb {Y}_{\theta _{2}}^{-1}(\pi ^{\mathbb {Y}}_{\theta _{2}1}(\theta _{1},\upsilon ))] \right] . \end{aligned} \end{aligned}$$

This implies

48 $$\begin{aligned} \begin{aligned}&\mathbb {Y}_{\theta _{2}}\mathcal {R}es_{1}\,\,\pi (\theta _{1},\upsilon )= \upsilon ^{\eta +1}\left[ g_{1}(\theta _{1})+\pi ^{2}(\theta _{1})\frac{\partial }{\partial \theta _{1}}\pi (\theta _{1})+\lambda \frac{\partial ^{3}}{\partial \theta _{1}^{3}}\pi (\theta _{1})+\lambda '\frac{\partial ^{5}}{\partial \theta _{1}^{5}}\pi (\theta _{1})\right] \\&+\upsilon ^{2\eta +1}\left[ \pi ^{2}(\theta _{1})\frac{\partial }{\partial \theta _{1}}g_{1}(\theta _{1})+2\pi (\theta _{1})g_{1}(\theta _{1})\frac{\partial }{\partial \theta _{1}}\pi (\theta _{1})+\lambda \frac{\partial ^{3}}{\partial \theta _{1}^{3}}g_{1}(\theta _{1})+\lambda '\frac{\partial ^{5}}{\partial \theta _{1}^{5}}g_{1}(\theta _{1})\right] \\&+\upsilon ^{3\eta +1}\Big [\dfrac{2\Gamma (2\eta +1)}{(\Gamma (\eta +1))^{2}}\pi (\theta _{1})g_{1}(\theta _{1})\frac{\partial }{\partial \theta _{1}}g_{1}(\theta _{1}) + \dfrac{\Gamma (2\eta +1)}{(\Gamma (\eta +1))^{2}}h^{2}_{1}(\theta _{1})\frac{\partial }{\partial \theta _{1}}\pi (\theta _{1})\Big ]\\&+ \upsilon ^{4\eta +1}\dfrac{\Gamma (3\eta +1)}{(\Gamma (\eta +1))^{3}}h^{2}_{1}(\theta _{1})\frac{\partial }{\partial \theta _{1}}g_{1}(\theta _{1}) \end{aligned} \end{aligned}$$

where $\pi (\theta _{1})=\frac{1}{\sqrt{-\lambda '}}[0.9487\lambda +0.0474\frac{\lambda ^{2}}{\lambda '} \theta _{1}^{2}+0.0021\frac{\lambda ^{3}}{\lambda '^{2}} \theta _{1}^{4}+0.3584\frac{\lambda ^{4}}{\lambda '^{3}} \theta _{1}^{6}]$. By multiplying (48) by $\upsilon ^{\eta +1}$ and by (47) we get

49 $$\begin{aligned} \begin{aligned}&g_{1}(\theta _{1})= \frac{0.0899\lambda ^{3}\sqrt{-\lambda '}+258.0984\lambda ^{4}}{\lambda '^{2}\sqrt{-\lambda '}} \theta _{1}+\frac{0.0125\lambda ^{3}\sqrt{-\lambda '}+43.008\lambda ^{5}}{\lambda '^{3}\sqrt{-\lambda '}} \theta _{1}^{3}\\&+\frac{2.0406\lambda ^{5}}{\lambda '^{4}} \theta _{1}^{5}+\frac{0.136\lambda ^{6}}{\lambda '^{5}} \theta _{1}^{7}+\frac{0.008\lambda ^{7}}{\lambda '^{6}} \theta _{1}^{9}+\frac{0.771\lambda ^{8}}{\lambda '^{7}} \theta _{1}^{11}. \end{aligned} \end{aligned}$$

At *n=2*, by (43)

$$\begin{aligned} \pi ^{\mathbb {Y}}_{\theta _{2}2}(\theta _{1},\upsilon )= \frac{\upsilon }{\sqrt{-\lambda '}}[0.9487\lambda +0.0474\frac{\lambda ^{2}}{\lambda '} \theta _{1}^{2}+0.0021\frac{\lambda ^{3}}{\lambda '^{2}} \theta _{1}^{4}+0.3584\frac{\lambda ^{4}}{\lambda '^{3}} \theta _{1}^{6}]+\upsilon ^{\eta +1}g_{1}(\theta _{1}) +\upsilon ^{2\eta +1}g_{2}(\theta _{1}) \end{aligned}$$

and (46) becomes

$$\begin{aligned} \begin{aligned}&\mathbb {Y}_{\theta _{2}}\mathcal {R}es_{2}\,\,\pi (\theta _{1},\upsilon )= \upsilon ^{\eta +1}g_{1}(\theta _{1}) +\upsilon ^{2\eta +1}g_{2}(\theta _{1}) +\upsilon ^{\eta }\mathbb {Y}_{\theta _{2}}\left[ [\mathbb {Y}_{\theta _{2}}^{-1}(\pi ^{\mathbb {Y}}_{\theta _{2}2}(\theta _{1},\upsilon ))]^{2}\frac{\partial }{\partial \theta _{1}}[\mathbb {Y}_{\theta _{2}}^{-1}(\pi ^{\mathbb {Y}}_{\theta _{2}2}(\theta _{1},\upsilon ))]\right] \\&+\lambda \upsilon ^{\eta }\mathbb {Y}_{\theta _{2}}\left[ \frac{\partial ^{3}}{\partial \theta _{1}^{3}}[\mathbb {Y}_{\theta _{2}}^{-1}(\pi ^{\mathbb {Y}}_{\theta _{2}2}(\theta _{1},\upsilon ))]\right] +\upsilon ^{\eta }\lambda '\mathbb {Y}_{\theta _{2}}\left[ \frac{\partial ^{5}}{\partial \theta _{1}^{5}}[\mathbb {Y}_{\theta _{2}}^{-1}(\pi ^{\mathbb {Y}}_{\theta _{2}2}(\theta _{1},\upsilon ))] \right] . \end{aligned} \end{aligned}$$

This implies

50 $$\begin{aligned} \begin{aligned}&\mathbb {Y}_{\theta _{2}}\mathcal {R}es_{2}\,\,\pi (\theta _{1},\upsilon )= \upsilon ^{2\eta +1}\Big [g_{2}(\theta _{1})+2\pi ^{2}(\theta _{1})\frac{\partial }{\partial \theta _{1}}g_{1}(\theta _{1})+2g_{1}(\theta _{1})\frac{\partial }{\partial \theta _{1}}\pi (\theta _{1})\\&+\lambda \frac{\partial ^{3}}{\partial \theta _{1}^{3}}g_{1}(\theta _{1})+\lambda '\frac{\partial ^{5}}{\partial \theta _{1}^{5}}g_{1}(\theta _{1})\Big ]+\upsilon ^{3\eta +1}\Big [\pi ^{2}(\theta _{1})\frac{\partial }{\partial \theta _{1}}g_{2}(\theta _{1})\\ &+ \frac{2\Gamma (2\eta +1)}{(\Gamma (\eta +1))^{2}}\pi (\theta _{1})g_{1}(\theta _{1})\frac{\partial }{\partial \theta _{1}}g_{1}(\theta _{1})+2g_{2}(\theta _{1})\pi (\theta _{1})\frac{\partial }{\partial \theta _{1}}\pi (\theta _{1})\\ &+g_{1}^{2}(\theta _{1})\frac{\partial }{\partial \theta _{1}}\pi (\theta _{1})+\lambda \frac{\partial ^{3}}{\partial \theta _{1}^{3}}g_{2}(\theta _{1})+\lambda '\frac{\partial ^{5}}{\partial \theta _{1}^{5}}g_{2}(\theta _{1})\Big ]\\&+\upsilon ^{4\eta +1}\Big [ \frac{2\Gamma (3\eta +1)}{\Gamma (\eta +1)\Gamma (2\eta +1)}\pi (\theta _{1})g_{2}(\theta _{1})\frac{\partial }{\partial \theta _{1}}g_{1}(\theta _{1})\\ &+\frac{2\Gamma (3\eta +1)}{\Gamma (2\eta +1)}\pi (\theta _{1})g_{1}(\theta _{1})\frac{\partial }{\partial \theta _{1}}g_{2}(\theta _{1})+\frac{\Gamma (3\eta +1)}{(\Gamma (\eta +1))^{3}}h^{2}_{1}(\theta _{1})\frac{\partial }{\partial \theta _{1}}g_{1}(\theta _{1}) \Big ]\\&+\upsilon ^{5\eta +1}\Big [ \frac{2\Gamma (4\eta +1)}{(\Gamma (2\eta +1))^{2}}\pi (\theta _{1})g_{2}(\theta _{1})\frac{\partial }{\partial \theta _{1}}g_{2}(\theta _{1})\\ &+\frac{\Gamma (4\eta +1)}{(\Gamma (\eta +1))^{2}\Gamma (2\eta +1)}h^{2}_{1}(\theta _{1})\frac{\partial }{\partial \theta _{1}}g_{2}(\theta _{1})\\ &+\frac{2\Gamma (4\eta +1)}{\Gamma (\eta +1) (\Gamma (2\eta +1))^{2}}g_{1}(\theta _{1})g_{2}(\theta _{1})\frac{\partial }{\partial \theta _{1}}\pi (\theta _{1}) \\ &+\frac{2\Gamma (4\eta +1)}{ (\Gamma (\eta +1))^{2}\Gamma (2\eta +1)}g_{1}(\theta _{1})g_{2}(\theta _{1})\frac{\partial }{\partial \theta _{1}}g_{1}(\theta _{1}) \\ &+\frac{\Gamma (4\eta +1)}{\Gamma (\eta +1) (\Gamma (2\eta +1))^{2}}h^{2}_{2}(\theta _{1})\frac{\partial }{\partial \theta _{1}}g_{1}(\theta _{1}) \Big ]\\&+ \upsilon ^{6\eta +1} \Big [ \frac{2\Gamma (5\eta +1)}{\Gamma (\eta +1)(\Gamma (2\eta +1))^{2}}g_{1}(\theta _{1})g_{2}(\theta _{1})\frac{\partial }{\partial \theta _{1}}g_{2}(\theta _{1})\\ &+ \frac{\Gamma (5\eta +1)}{\Gamma (\eta +1)(\Gamma (2\eta +1))^{2}}h^{2}_{2}(\theta _{1})\frac{\partial }{\partial \theta _{1}}g_{1}(\theta _{1})\Big ]\\&+ \upsilon ^{7\eta +1} \frac{\Gamma (6\eta +1)}{(\Gamma (2\eta +1))^{3}}h^{2}_{2}(\theta _{1}) \frac{\partial }{\partial \theta _{1}}g_{2}(\theta _{1})\Big ]. \end{aligned} \end{aligned}$$

By multiplying (50) by $\upsilon ^{-\eta +1}$ and by (47) we have

51 $$\begin{aligned} \begin{aligned}&g_{2}(\theta _{1})=-2\pi ^{2}(\theta _{1})\frac{\partial }{\partial \theta _{1}}g_{1}(\theta _{1})-2g_{1}(\theta _{1})\frac{\partial }{\partial \theta _{1}}\pi (\theta _{1})-\lambda \frac{\partial ^{3}}{\partial \theta _{1}^{3}}g_{1}(\theta _{1})-\lambda '\frac{\partial ^{5}}{\partial \theta _{1}^{5}}g_{1}(\theta _{1}). \end{aligned} \end{aligned}$$

Put the terms of $\left<g_{n}\right>_{n\in \mathbb {N}\cup \{0\}}$ in (36) to get

52 $$\begin{aligned} \begin{aligned}&\pi ^{\mathbb {Y}}_{\theta _{2}}(\theta _{1},\upsilon )=\sum _{j=0}^{\infty }\upsilon ^{j\eta +1}g_{j}(\theta _{1})= \upsilon g_{0}(\theta _{1})+\upsilon ^{\eta +1} g_{1}(\theta _{1})+\upsilon ^{2\eta +1} g_{2}(\theta _{1})+\cdots \\&= \upsilon \Big [0.9487\frac{\lambda }{\sqrt{-\lambda '}}+0.0474\frac{\lambda ^{2}}{\lambda '\sqrt{-\lambda '}} \theta _{1}^{2}+0.0021\frac{\lambda ^{3}}{\lambda '^{2}\sqrt{-\lambda '}} \theta _{1}^{4}+0.3584\frac{\lambda ^{4}}{\lambda '^{3}\sqrt{-\lambda '}} \theta _{1}^{6}\Big ] \\ &+\upsilon ^{\eta +1} \Big \{\frac{0.0899\lambda ^{3}\sqrt{-\lambda '}+258.0984\lambda ^{4}}{\lambda '^{2}\sqrt{-\lambda '}} \theta _{1}+\frac{0.0125\lambda ^{3}\sqrt{-\lambda '}+43.008\lambda ^{5}}{\lambda '^{3}\sqrt{-\lambda '}} \theta _{1}^{3}\\&+\frac{2.0406\lambda ^{5}}{\lambda '^{4}} \theta _{1}^{5}+\frac{0.136\lambda ^{6}}{\lambda '^{5}} \theta _{1}^{7}+\frac{0.008\lambda ^{7}}{\lambda '^{6}} \theta _{1}^{9}+\frac{0.771\lambda ^{8}}{\lambda '^{7}} \theta _{1}^{11}\Big \}\\&-\upsilon ^{2\eta +1}\Big [2\pi ^{2}(\theta _{1})\frac{\partial }{\partial \theta _{1}}g_{1}(\theta _{1})+2g_{1}(\theta _{1})\frac{\partial }{\partial \theta _{1}}\pi (\theta _{1})+\lambda \frac{\partial ^{3}}{\partial \theta _{1}^{3}}g_{1}(\theta _{1})+\\&\lambda '\frac{\partial ^{5}}{\partial \theta _{1}^{5}}g_{1}(\theta _{1})\Big ] +\cdots \end{aligned} \end{aligned}$$

Now take the inverse Yang transform of (52)

53 $$\begin{aligned} \begin{aligned}&\pi (\theta _{1},\theta _{2})=0.9487\frac{\lambda }{\sqrt{-\lambda '}}+0.0474\frac{\lambda ^{2}}{\lambda '\sqrt{-\lambda '}} \theta _{1}^{2}+0.0021\frac{\lambda ^{3}}{\lambda '^{2}\sqrt{-\lambda '}} \theta _{1}^{4}+0.3584\frac{\lambda ^{4}}{\lambda '^{3}\sqrt{-\lambda '}} \theta _{1}^{6} \\ &+\dfrac{\theta _{2}^{\eta }}{\Gamma (\eta +1)} \Big \{\frac{0.0899\lambda ^{3}\sqrt{-\lambda '}+258.0984\lambda ^{4}}{\lambda '^{2}\sqrt{-\lambda '}} \theta _{1}+\frac{0.0125\lambda ^{3}\sqrt{-\lambda '}+43.008\lambda ^{5}}{\lambda '^{3}\sqrt{-\lambda '}} \theta _{1}^{3}\\&+\frac{2.0406\lambda ^{5}}{\lambda '^{4}} \theta _{1}^{5}+\frac{0.136\lambda ^{6}}{\lambda '^{5}} \theta _{1}^{7}+\frac{0.008\lambda ^{7}}{\lambda '^{6}} \theta _{1}^{9}+\frac{0.771\lambda ^{8}}{\lambda '^{7}} \theta _{1}^{11}\Big \}\\&-\dfrac{\theta _{2}^{2\eta }}{\Gamma (2\eta +1)}\Big [2\pi ^{2}(\theta _{1})\frac{\partial }{\partial \theta _{1}}g_{1}(\theta _{1})+2g_{1}(\theta _{1})\frac{\partial }{\partial \theta _{1}}\pi (\theta _{1})+\lambda \frac{\partial ^{3}}{\partial \theta _{1}^{3}}g_{1}(\theta _{1})+\\&\lambda '\frac{\partial ^{5}}{\partial \theta _{1}^{5}}g_{1}(\theta _{1})\Big ] +\cdots \end{aligned} \end{aligned}$$

Secondly, with the exact initial conditions. Consider the following MCFKE

54 $$\begin{aligned} \left\{ \begin{aligned}&{}^{c}\mathcal {D}_{0,\theta _{2}}^{\eta }\pi (\theta _{1},\theta _{2})+\pi ^{\mathbb {Y}2}(\theta _{1},\theta _{2})\frac{\partial \pi (\theta _{1},\theta _{2})}{\partial \theta _{1}}+\lambda \frac{\partial ^{3} \pi (\theta _{1},\theta _{2})}{\partial \theta _{1}^{3}}+\lambda '\frac{\partial ^{5} \pi (\theta _{1},\theta _{2})}{\partial \theta _{1}^{5}}=0,\,\,\,\,\,\,\,\,0<\eta \le 1\\&\pi (\theta _{1},0)=\frac{3 \lambda }{\sqrt{-10 \lambda '}}\,\,\text{ sech}^{2}\left( \frac{1}{2}\sqrt{\frac{-\lambda }{5\lambda '}}\,\, \theta _{1}\right) \end{aligned} \right. \end{aligned}$$

where *λ >0*, *λ '<0* are real numbers. By using^36^ that if *η =1* then the exact solution of (54) is

55 $$\begin{aligned} \pi (\theta _{1},\theta _{2})=\frac{3 \lambda }{\sqrt{-10 \lambda '}}\,\,\text{ sech}^{2}\left[ \frac{1}{2}\sqrt{\frac{-\lambda }{5\lambda '}} \left( \theta _{1}-\frac{25\lambda '-4\lambda ^{2}}{25\lambda '} \,\,\theta _{2}\right) \right] . \end{aligned}$$

Take the Yang transforms on (54)

56 $$\begin{aligned} \begin{aligned}&\pi ^{\mathbb {Y}}_{\theta _{2}}(\theta _{1},\upsilon )=\frac{3 \lambda }{\upsilon \sqrt{-10 \lambda '}}\,\,\text{ sech}^{2}\left( \frac{1}{2}\sqrt{\frac{-\lambda }{5\lambda '}}\,\, \theta _{1}\right) \\ &-\upsilon ^{\eta }\mathbb {Y}_{\theta _{2}}\left[ \Big [\mathbb {Y}_{\theta _{2}}^{-1}(\pi ^{\mathbb {Y}}_{\theta _{2}}(\theta _{1},\upsilon ))\Big ]^{2}\frac{\partial }{\partial \theta _{1}}[\mathbb {Y}_{\theta _{2}}^{-1}(\pi ^{\mathbb {Y}}_{\theta _{2}}(\theta _{1},\upsilon ))]\right] \\&-\lambda \upsilon ^{\eta }\mathbb {Y}_{\theta _{2}}\left[ \frac{\partial ^{3}}{\partial \theta _{1}^{3}}[\mathbb {Y}_{\theta _{2}}^{-1}(\pi ^{\mathbb {Y}}_{\theta _{2}}(\theta _{1},\upsilon ))]\right] -\upsilon ^{\eta }\lambda '\mathbb {Y}_{\theta _{2}}\left[ \frac{\partial ^{5}}{\partial \theta _{1}^{5}}[\mathbb {Y}_{\theta _{2}}^{-1}(\pi ^{\mathbb {Y}}_{\theta _{2}}(\theta _{1},\upsilon ))] \right] \end{aligned} \end{aligned}$$

with the sequence $\left<\pi ^{\mathbb {Y}}_{ \theta _{2} n}\right>_{n\in \mathbb {N}\cup \{0\}}$

57 $$\begin{aligned} \pi ^{\mathbb {Y}}_{ \theta _{2} n}(\theta _{1},\upsilon )=\frac{3 \lambda \upsilon }{\sqrt{-10 \lambda '}}\,\,\text{ sech}^{2}\left( \frac{1}{2}\sqrt{\frac{-\lambda }{5\lambda '}}\,\, \theta _{1}\right) +\sum _{j=1}^{n}\upsilon ^{j\eta +1}g_{j}(\theta _{1}). \end{aligned}$$

The *n*-th structure for (56) is

58 $$\begin{aligned} \begin{aligned}&\pi ^{\mathbb {Y}}_{ \theta _{2} n}(\theta _{1},\upsilon )=\frac{3 \lambda \upsilon }{\sqrt{-10 \lambda '}}\,\,\text{ sech}^{2}\left( \frac{1}{2}\sqrt{\frac{-\lambda }{5\lambda '}}\,\, \theta _{1}\right) \\ &-\upsilon ^{\eta }\mathbb {Y}_{\theta _{2}}\left[ \Big [\mathbb {Y}_{\theta _{2}}^{-1}(\pi ^{\mathbb {Y}}_{ \theta _{2} n}(\theta _{1},\upsilon ))\Big ]^{2}\frac{\partial }{\partial \theta _{1}}[\mathbb {Y}_{\theta _{2}}^{-1}(\pi ^{\mathbb {Y}}_{ \theta _{2} n}(\theta _{1},\upsilon ))]\right] \\&-\lambda \upsilon ^{\eta }\mathbb {Y}_{\theta _{2}}\left[ \frac{\partial ^{3}}{\partial \theta _{1}^{3}}[\mathbb {Y}_{\theta _{2}}^{-1}(\pi ^{\mathbb {Y}}_{ \theta _{2} n}(\theta _{1},\upsilon ))]\right] -\upsilon ^{\eta }\lambda '\mathbb {Y}_{\theta _{2}}\left[ \frac{\partial ^{5}}{\partial \theta _{1}^{5}}[\mathbb {Y}_{\theta _{2}}^{-1}(\pi ^{\mathbb {Y}}_{ \theta _{2} n}(\theta _{1},\upsilon ))] \right] . \end{aligned} \end{aligned}$$

The Yang residual map, $\mathbb {Y}_{\theta _{2}}\mathcal {R}es\,\,\pi (\theta _{1},\upsilon )$ for (56) is given by

59 $$\begin{aligned} \begin{aligned}&\mathbb {Y}_{\theta _{2}}\mathcal {R}es\,\,\pi (\theta _{1},\upsilon )= \pi ^{\mathbb {Y}}_{\theta _{2}}(\theta _{1},\upsilon )-\frac{3 \lambda \upsilon }{\sqrt{-10 \lambda '}}\,\,\text{ sech}^{2}\left( \frac{1}{2}\sqrt{\frac{-\lambda }{5\lambda '}}\,\, \theta _{1}\right) \\&+\upsilon ^{\eta }\mathbb {Y}_{\theta _{2}}\left[ \Big [\mathbb {Y}_{\theta _{2}}^{-1}(\pi ^{\mathbb {Y}}_{\theta _{2}}(\theta _{1},\upsilon ))\Big ]^{2}\frac{\partial }{\partial \theta _{1}}[\mathbb {Y}_{\theta _{2}}^{-1}(\pi ^{\mathbb {Y}}_{\theta _{2}}(\theta _{1},\upsilon ))]\right] \\&+\lambda \upsilon ^{\eta }\mathbb {Y}_{\theta _{2}}\left[ \frac{\partial ^{3}}{\partial \theta _{1}^{3}}[\mathbb {Y}_{\theta _{2}}^{-1}(\pi ^{\mathbb {Y}}_{\theta _{2}}(\theta _{1},\upsilon ))]\right] +\upsilon ^{\eta }\lambda '\mathbb {Y}_{\theta _{2}}\left[ \frac{\partial ^{5}}{\partial \theta _{1}^{5}}[\mathbb {Y}_{\theta _{2}}^{-1}(\pi ^{\mathbb {Y}}_{\theta _{2}}(\theta _{1},\upsilon ))] \right] \end{aligned} \end{aligned}$$

with the *n*-th structure

60 $$\begin{aligned} \begin{aligned}&\mathbb {Y}_{\theta _{2}}\mathcal {R}es_{n}\,\,\pi (\theta _{1},\upsilon )= \pi ^{\mathbb {Y}}_{ \theta _{2} n}(\theta _{1},\upsilon )-\frac{3 \lambda \upsilon }{\sqrt{-10 \lambda '}}\,\,\text{ sech}^{2}\left( \frac{1}{2}\sqrt{\frac{-\lambda }{5\lambda '}}\,\, \theta _{1}\right) \\&+\upsilon ^{\eta }\mathbb {Y}_{\theta _{2}}\left[ \Big [\mathbb {Y}_{\theta _{2}}^{-1}(\pi ^{\mathbb {Y}}_{ \theta _{2} n}(\theta _{1},\upsilon ))\Big ]^{2}\frac{\partial }{\partial \theta _{1}}[\mathbb {Y}_{\theta _{2}}^{-1}(\pi ^{\mathbb {Y}}_{ \theta _{2} n}(\theta _{1},\upsilon ))]\right] \\&+\lambda \upsilon ^{\eta }\mathbb {Y}_{\theta _{2}}\left[ \frac{\partial ^{3}}{\partial \theta _{1}^{3}}[\mathbb {Y}_{\theta _{2}}^{-1}(\pi ^{\mathbb {Y}}_{ \theta _{2} n}(\theta _{1},\upsilon ))]\right] +\upsilon ^{\eta }\lambda '\mathbb {Y}_{\theta _{2}}\left[ \frac{\partial ^{5}}{\partial \theta _{1}^{5}}[\mathbb {Y}_{\theta _{2}}^{-1}(\pi ^{\mathbb {Y}}_{ \theta _{2} n}(\theta _{1},\upsilon ))] \right] \end{aligned} \end{aligned}$$

for *n=1,2,3,⋯*. The terms of $\left<g_{n}\right>_{n\in \mathbb {N}\cup \{0\}}$ are calculating by the relation

61 $$\begin{aligned} \lim _{\upsilon \rightarrow 0}\upsilon ^{-n\eta -1}\mathbb {Y}_{\theta _{2}}\mathcal {R}es_{n}\,\,\pi (\theta _{1},\upsilon )=0\,\,\,\,\,\,\,\, n=1,2,3,\cdots \end{aligned}$$

At *n=1*, by (57) we have $\pi ^{\mathbb {Y}}_{\theta _{2}1}(\theta _{1},\upsilon )= \frac{3 \lambda \upsilon }{\sqrt{-10 \lambda '}}\,\,\text{ sech}^{2}\left( \frac{1}{2}\sqrt{\frac{-\lambda }{5\lambda '}}\,\, \theta _{1}\right) +\upsilon ^{\eta +1}g_{1}(\theta _{1})$ and (60) becomes

$$\begin{aligned} \begin{aligned}&\mathbb {Y}_{\theta _{2}}\mathcal {R}es_{1}\,\,\pi (\theta _{1},\upsilon )= \upsilon ^{\eta +1}g_{1}(\theta _{1}) +\upsilon ^{\eta }\mathbb {Y}_{\theta _{2}}\left[ \Big [\mathbb {Y}_{\theta _{2}}^{-1}(\pi ^{\mathbb {Y}}_{\theta _{2}1}(\theta _{1},\upsilon ))\Big ]^{2}\frac{\partial }{\partial \theta _{1}}[\mathbb {Y}_{\theta _{2}}^{-1}(\pi ^{\mathbb {Y}}_{\theta _{2}1}(\theta _{1},\upsilon ))]\right] \\&+\lambda \upsilon ^{\eta }\mathbb {Y}_{\theta _{2}}\left[ \frac{\partial ^{3}}{\partial \theta _{1}^{3}}[\mathbb {Y}_{\theta _{2}}^{-1}(\pi ^{\mathbb {Y}}_{\theta _{2}1}(\theta _{1},\upsilon ))]\right] +\upsilon ^{\eta }\lambda '\mathbb {Y}_{\theta _{2}}\left[ \frac{\partial ^{5}}{\partial \theta _{1}^{5}}[\mathbb {Y}_{\theta _{2}}^{-1}(\pi ^{\mathbb {Y}}_{\theta _{2}1}(\theta _{1},\upsilon ))] \right] . \end{aligned} \end{aligned}$$

This implies

62 $$\begin{aligned} \begin{aligned}&\mathbb {Y}_{\theta _{2}}\mathcal {R}es_{1}\,\,\pi (\theta _{1},\upsilon )= \upsilon ^{\eta +1}\left[ g_{1}(\theta _{1})+\mathcal {H}^{2}(\theta _{1})\frac{\partial }{\partial \theta _{1}}\mathcal {H}(\theta _{1})+\lambda \frac{\partial ^{3}}{\partial \theta _{1}^{3}}\mathcal {H}(\theta _{1})+\lambda '\frac{\partial ^{5}}{\partial \theta _{1}^{5}}\mathcal {H}(\theta _{1})\right] \\&+\upsilon ^{2\eta +1}\left[ \mathcal {H}^{2}(\theta _{1})\frac{\partial }{\partial \theta _{1}}g_{1}(\theta _{1})+2\mathcal {H}(\theta _{1})g_{1}(\theta _{1})\frac{\partial }{\partial \theta _{1}}\mathcal {H}(\theta _{1})+\lambda \frac{\partial ^{3}}{\partial \theta _{1}^{3}}g_{1}(\theta _{1})+\lambda '\frac{\partial ^{5}}{\partial \theta _{1}^{5}}g_{1}(\theta _{1})\right] \\&+ \upsilon ^{3\eta +1} \Big [\dfrac{2\Gamma (2\eta +1)}{(\Gamma (\eta +1))^{2}}\mathcal {H}(\theta _{1})g_{1}(\theta _{1})\frac{\partial }{\partial \theta _{1}}g_{1}(\theta _{1}) + \dfrac{\Gamma (2\eta +1)}{(\Gamma (\eta +1))^{2}}h^{2}_{1}(\theta _{1})\frac{\partial }{\partial \theta _{1}}\mathcal {H}(\theta _{1})\Big ]\\&+ \upsilon ^{4\eta +1} \dfrac{\Gamma (3\eta +1)}{(\Gamma (\eta +1))^{3}}h^{2}_{1}(\theta _{1})\frac{\partial }{\partial \theta _{1}}g_{1}(\theta _{1}) \end{aligned} \end{aligned}$$

where $\mathcal {H}(\theta _{1})=\frac{3 \lambda }{\sqrt{-10 \lambda '}}\,\,\text{ sech}^{2}\left( \frac{1}{2}\sqrt{\frac{-\lambda }{5\lambda '}}\,\, \theta _{1}\right)$. By multiplying (62) by $\upsilon ^{-\eta +1}$ and by (61) we have

63 $$\begin{aligned} \begin{aligned}&g_{1}(\theta _{1})= \frac{27\lambda ^{3}\sqrt{\lambda }}{5\lambda ' \sqrt{2}}\text{ sech}^6\left( \frac{1}{2}\sqrt{\frac{-\lambda }{5\lambda '}}\theta _{1}\right) \text{ tanh } \left( \frac{1}{2}\sqrt{\frac{-\lambda }{5\lambda '}}\theta _{1}\right) \\&+\frac{6\lambda ^{3}\sqrt{\lambda }}{25\lambda '^{2} \sqrt{2}}\text{ sech}^4\left( \frac{1}{2}\sqrt{\frac{-\lambda }{5\lambda '}}\theta _{1}\right) \text{ tanh } \left( \frac{1}{2}\sqrt{\frac{-\lambda }{5\lambda '}}\theta _{1}\right) -\frac{ \lambda }{ 5\lambda ' \sqrt{-10\lambda '}} \text{ tanh}^{2} \left( \frac{1}{2}\sqrt{\frac{-\lambda }{5\lambda '}}\theta _{1}\right) \\&-\frac{39\lambda ^{3}\sqrt{\lambda }}{125\lambda '^{3} \sqrt{2}}\text{ sech}^4\left( \frac{1}{2}\sqrt{\frac{-\lambda }{5\lambda '}}\theta _{1}\right) \text{ tanh}^{3} \left( \frac{1}{2}\sqrt{\frac{-\lambda }{5\lambda '}}\theta _{1}\right) \\&+\frac{51\lambda ^{3}\sqrt{\lambda }}{250\lambda '^{3} \sqrt{2}}\text{ sech}^6\left( \frac{1}{2}\sqrt{\frac{-\lambda }{5\lambda '}}\theta _{1}\right) \text{ tanh } \left( \frac{1}{2}\sqrt{\frac{-\lambda }{5\lambda '}}\theta _{1}\right) \\&+\frac{3\lambda ^{3}\sqrt{\lambda }}{125\lambda '^{3} \sqrt{2}}\text{ sech}^2\left( \frac{1}{2}\sqrt{\frac{-\lambda }{5\lambda '}}\theta _{1}\right) \text{ tanh}^{5} \left( \frac{1}{2}\sqrt{\frac{-\lambda }{5\lambda '}}\theta _{1}\right) . \end{aligned} \end{aligned}$$

At *n=2*, by (57)

$$\begin{aligned} \pi ^{\mathbb {Y}}_{\theta _{2}2}(\theta _{1},\upsilon )= \frac{3 \lambda \upsilon }{\sqrt{-10 \lambda '}}\,\,\text{ sech}^{2}\left( \frac{1}{2}\sqrt{\frac{-\lambda }{5\lambda '}}\,\, \theta _{1}\right) +\upsilon ^{\eta +1}g_{1}(\theta _{1}) +\upsilon ^{2\eta +1}g_{2}(\theta _{1}) . \end{aligned}$$

Then we have

$$\begin{aligned} \begin{aligned}&\mathbb {Y}_{\theta _{2}}\mathcal {R}es_{2}\,\,\pi (\theta _{1},\upsilon )= \upsilon ^{\eta +1}g_{1}(\theta _{1}) +\upsilon ^{2\eta +1}g_{2}(\theta _{1}) +\upsilon ^{\eta }\mathbb {Y}_{\theta _{2}}\left[ \Big [\mathbb {Y}_{\theta _{2}}^{-1}(\pi ^{\mathbb {Y}}_{\theta _{2}2}(\theta _{1},\upsilon ))\Big ]^{2}\frac{\partial }{\partial \theta _{1}}[\mathbb {Y}_{\theta _{2}}^{-1}(\pi ^{\mathbb {Y}}_{\theta _{2}2}(\theta _{1},\upsilon ))]\right] \\&+\lambda \upsilon ^{\eta }\mathbb {Y}_{\theta _{2}}\left[ \frac{\partial ^{3}}{\partial \theta _{1}^{3}}[\mathbb {Y}_{\theta _{2}}^{-1}(\pi ^{\mathbb {Y}}_{\theta _{2}2}(\theta _{1},\upsilon ))]\right] +\upsilon ^{\eta }\lambda '\mathbb {Y}_{\theta _{2}}\left[ \frac{\partial ^{5}}{\partial \theta _{1}^{5}}[\mathbb {Y}_{\theta _{2}}^{-1}(\pi ^{\mathbb {Y}}_{\theta _{2}2}(\theta _{1},\upsilon ))] \right] . \end{aligned} \end{aligned}$$

This implies

64 $$\begin{aligned} \begin{aligned}&\mathbb {Y}_{\theta _{2}}\mathcal {R}es_{2}\,\,\pi (\theta _{1},\upsilon )= \upsilon ^{2\eta +1}\Big [g_{2}(\theta _{1})+\mathcal {H}^{2}(\theta _{1})\frac{\partial }{\partial \theta _{1}}g_{1}(\theta _{1})+2g_{1}(\theta _{1})\mathcal {H}(\theta _{1})\dfrac{\partial }{\partial \theta _{1}}\mathcal {H}(\theta _{1})\\&+ \lambda \frac{\partial ^{3}}{\partial \theta _{1}^{3}}g_{1}(\theta _{1})+\lambda '\frac{\partial ^{5}}{\partial \theta _{1}^{5}}g_{1}(\theta _{1})\Big ]+\upsilon ^{3\eta +1}\Big [\mathcal {H}^{2}(\theta _{1})g_{2}^{2}(\theta _{1})\\&+\dfrac{2\Gamma (2\eta +1)}{(\Gamma (\eta +1))^{2}}\mathcal {H}(\theta _{1})g_{1}(\theta _{1})\frac{\partial }{\partial \theta _{1}}g_{1}(\theta _{1})+2g_{2}(\theta _{1})\mathcal {H}(\theta _{1})\frac{\partial }{\partial \theta _{1}}\mathcal {H}(\theta _{1})\\&+\dfrac{\Gamma (2\eta +1)}{(\Gamma (\eta +1))^{2}}h^{2}_{1}(\theta _{1})\frac{\partial }{\partial \theta _{1}}\mathcal {H}(\theta _{1})+\lambda \frac{\partial ^{3}}{\partial \theta _{1}^{3}}g_{2}(\theta _{1})+\lambda '\frac{\partial ^{5}}{\partial \theta _{1}^{5}}g_{2}(\theta _{1})\Big ]\\&+ \upsilon ^{4\eta +1} \Big [\dfrac{2\Gamma (3\eta +1)}{(\Gamma (2\eta +1))^{2}}\mathcal {H}(\theta _{1})g_{1}(\theta _{1})\frac{\partial }{\partial \theta _{1}}g_{1}(\theta _{1})\\&+ \dfrac{2\Gamma (3\eta +1)}{\Gamma (\eta +1)\Gamma (2\eta +1)}g_{2}(\theta _{1})\mathcal {H}(\theta _{1})\frac{\partial }{\partial \theta _{1}}g_{1}(\theta _{1})\\&+ \dfrac{\Gamma (3\eta +1)}{(\Gamma (\eta +1))^{3}}h^{2}_{1}(\theta _{1})\frac{\partial }{\partial \theta _{1}}g_{1}(\theta _{1}) + \dfrac{2\Gamma (3\eta +1)}{\Gamma (\eta +1)\Gamma (2\eta +1)}g_{1}(\theta _{1})g_{2}(\theta _{1})\frac{\partial }{\partial \theta _{1}}\mathcal {H}(\theta _{1})\Big ]\\&+\upsilon ^{5\eta +1}\Big [ \dfrac{2\Gamma (4\eta +1)}{(\Gamma (\eta +1))^{2}}\mathcal {H}(\theta _{1})g_{2}(\theta _{1})\frac{\partial }{\partial \theta _{1}}g_{2}(\theta _{1})+ \dfrac{\Gamma (4\eta +1)}{\Gamma (\eta +1)\Gamma (2\eta +1)}h^{2}_{1}(\theta _{1})\frac{\partial }{\partial \theta _{1}}g_{2}(\theta _{1})\\&+\dfrac{2\Gamma (4\eta +1)}{(\Gamma (\eta +1))^{2}\Gamma (2\eta +1)}g_{1}(\theta _{1})g_{2}(\theta _{1})\frac{\partial }{\partial \theta _{1}}g_{1}(\theta _{1})\\&+\dfrac{\Gamma (4\eta +1)}{(\Gamma (\eta +1))^{2}\Gamma (2\eta +1)}h^{2}_{2}(\theta _{1})\frac{\partial }{\partial \theta _{1}}\mathcal {H}(\theta _{1})\Big ]\\&+\upsilon ^{6\eta +1}\Big [ \dfrac{2\Gamma (5\eta +1)}{(\Gamma (2\eta +1))^{2}\Gamma (\eta +1)}g_{1}(\theta _{1})g_{2}(\theta _{1})\frac{\partial }{\partial \theta _{1}}g_{2}(\theta _{1})\\&+\dfrac{\Gamma (5\eta +1)}{(\Gamma (2\eta +1))^{2}\Gamma (\eta +1)}h^{2}_{2}(\theta _{1})\frac{\partial }{\partial \theta _{1}}g_{1}(\theta _{1})\Big ]+ \upsilon ^{7\eta +1} \dfrac{\Gamma (6\eta +1)}{(\Gamma (2\eta +1))^{3}}h^{2}_{2}(\theta _{1})\frac{\partial }{\partial \theta _{1}}g_{2}(\theta _{1}). \end{aligned} \end{aligned}$$

By multiplying (64) by $\upsilon ^{-\eta +1}$ and by (61) we have

65 $$\begin{aligned} \begin{aligned} g_{2}(\theta _{1})=-\mathcal {H}^{2}(\theta _{1})\frac{\partial }{\partial \theta _{1}}g_{1}(\theta _{1})-2g_{1}(\theta _{1})\mathcal {H}(\theta _{1})\dfrac{\partial }{\partial \theta _{1}}\mathcal {H}(\theta _{1})- \lambda \frac{\partial ^{3}}{\partial \theta _{1}^{3}}g_{1}(\theta _{1})-\lambda '\frac{\partial ^{5}}{\partial \theta _{1}^{5}}g_{1}(\theta _{1}). \end{aligned} \end{aligned}$$

Put the terms of $\left<g_{n}\right>_{n\in \mathbb {N}\cup \{0\}}$ in (36)

66 $$\begin{aligned} \begin{aligned}&\pi ^{\mathbb {Y}}_{\theta _{2}}(\theta _{1},\upsilon )=\sum _{j=0}^{\infty }\upsilon ^{j\eta +1}g_{j}(\theta _{1})= \upsilon g_{0}(\theta _{1})+\upsilon ^{\eta +1} g_{1}(\theta _{1})+\upsilon ^{2\eta +1} g_{2}(\theta _{1})+\cdots \\&= \frac{3 \lambda \upsilon }{\sqrt{-10 \lambda '}}\,\,\text{ sech}^{2}\left( \frac{1}{2}\sqrt{\frac{-\lambda }{5\lambda '}}\,\, \theta _{1}\right) \\ &+\upsilon ^{\eta +1} \Big \{\frac{27\lambda ^{3}\sqrt{\lambda }}{5\lambda ' \sqrt{2}}\text{ sech}^6\left( \frac{1}{2}\sqrt{\frac{-\lambda }{5\lambda '}}\theta _{1}\right) \text{ tanh } \left( \frac{1}{2}\sqrt{\frac{-\lambda }{5\lambda '}}\theta _{1}\right) \\&+\frac{6\lambda ^{3}\sqrt{\lambda }}{25\lambda '^{2} \sqrt{2}}\text{ sech}^4\left( \frac{1}{2}\sqrt{\frac{-\lambda }{5\lambda '}}\theta _{1}\right) \text{ tanh } \left( \frac{1}{2}\sqrt{\frac{-\lambda }{5\lambda '}}\theta _{1}\right) -\frac{ \lambda }{ 5\lambda ' \sqrt{-10\lambda '}} \text{ tanh}^{2} \left( \frac{1}{2}\sqrt{\frac{-\lambda }{5\lambda '}}\theta _{1}\right) \\&-\frac{39\lambda ^{3}\sqrt{\lambda }}{125\lambda '^{3} \sqrt{2}}\text{ sech}^4\left( \frac{1}{2}\sqrt{\frac{-\lambda }{5\lambda '}}\theta _{1}\right) \text{ tanh}^{3} \left( \frac{1}{2}\sqrt{\frac{-\lambda }{5\lambda '}}\theta _{1}\right) \\&+\frac{51\lambda ^{3}\sqrt{\lambda }}{250\lambda '^{3} \sqrt{2}}\text{ sech}^6\left( \frac{1}{2}\sqrt{\frac{-\lambda }{5\lambda '}}\theta _{1}\right) \text{ tanh } \left( \frac{1}{2}\sqrt{\frac{-\lambda }{5\lambda '}}\theta _{1}\right) \\&+\frac{3\lambda ^{3}\sqrt{\lambda }}{125\lambda '^{3} \sqrt{2}}\text{ sech}^2\left( \frac{1}{2}\sqrt{\frac{-\lambda }{5\lambda '}}\theta _{1}\right) \text{ tanh}^{5} \left( \frac{1}{2}\sqrt{\frac{-\lambda }{5\lambda '}}\theta _{1}\right) \Big \}-\upsilon ^{2\eta +1}\Big (\mathcal {H}^{2}(\theta _{1})\frac{\partial }{\partial \theta _{1}}g_{1}(\theta _{1})\\&+2g_{1}(\theta _{1})\mathcal {H}(\theta _{1})\dfrac{\partial }{\partial \theta _{1}}\mathcal {H}(\theta _{1}) \lambda \frac{\partial ^{3}}{\partial \theta _{1}^{3}}g_{1}(\theta _{1})+\lambda '\frac{\partial ^{5}}{\partial \theta _{1}^{5}}g_{1}(\theta _{1}))\Big )+\cdots \end{aligned} \end{aligned}$$

Take the inverse Yang transform of (66),

67 $$\begin{aligned} \begin{aligned}&\pi (\theta _{1},\theta _{2})=\frac{3 \lambda }{\sqrt{-10 \lambda '}}\,\,\text{ sech}^{2}\left( \frac{1}{2}\sqrt{\frac{-\lambda }{5\lambda '}}\,\, \theta _{1}\right) \\ &+\dfrac{\theta _{2}^{\eta }}{\Gamma (\eta +1)} \Big \{\frac{27\lambda ^{3}\sqrt{\lambda }}{5\lambda ' \sqrt{2}}\text{ sech}^6\left( \frac{1}{2}\sqrt{\frac{-\lambda }{5\lambda '}}\theta _{1}\right) \text{ tanh } \left( \frac{1}{2}\sqrt{\frac{-\lambda }{5\lambda '}}\theta _{1}\right) \\&+\frac{6\lambda ^{3}\sqrt{\lambda }}{25\lambda '^{2} \sqrt{2}}\text{ sech}^4\left( \frac{1}{2}\sqrt{\frac{-\lambda }{5\lambda '}}\theta _{1}\right) \text{ tanh } \left( \frac{1}{2}\sqrt{\frac{-\lambda }{5\lambda '}}\theta _{1}\right) -\frac{ \lambda }{ 5\lambda ' \sqrt{-10\lambda '}} \text{ tanh}^{2} \left( \frac{1}{2}\sqrt{\frac{-\lambda }{5\lambda '}}\theta _{1}\right) \\&-\frac{39\lambda ^{3}\sqrt{\lambda }}{125\lambda '^{3} \sqrt{2}}\text{ sech}^4\left( \frac{1}{2}\sqrt{\frac{-\lambda }{5\lambda '}}\theta _{1}\right) \text{ tanh}^{3} \left( \frac{1}{2}\sqrt{\frac{-\lambda }{5\lambda '}}\theta _{1}\right) \\&+\frac{51\lambda ^{3}\sqrt{\lambda }}{250\lambda '^{3} \sqrt{2}}\text{ sech}^6\left( \frac{1}{2}\sqrt{\frac{-\lambda }{5\lambda '}}\theta _{1}\right) \text{ tanh } \left( \frac{1}{2}\sqrt{\frac{-\lambda }{5\lambda '}}\theta _{1}\right) \\&+\frac{3\lambda ^{3}\sqrt{\lambda }}{125\lambda '^{3} \sqrt{2}}\text{ sech}^2\left( \frac{1}{2}\sqrt{\frac{-\lambda }{5\lambda '}}\theta _{1}\right) \text{ tanh}^{5} \left( \frac{1}{2}\sqrt{\frac{-\lambda }{5\lambda '}}\theta _{1}\right) \Big \}-\dfrac{\theta _{2}^{2\eta }}{\Gamma (2\eta +1)}\Big (\mathcal {H}^{2}(\theta _{1})\frac{\partial }{\partial \theta _{1}}g_{1}(\theta _{1})\\&+2g_{1}(\theta _{1})\mathcal {H}(\theta _{1})\dfrac{\partial }{\partial \theta _{1}}\mathcal {H}(\theta _{1}) \lambda \frac{\partial ^{3}}{\partial \theta _{1}^{3}}g_{1}(\theta _{1})+\lambda '\frac{\partial ^{5}}{\partial \theta _{1}^{5}}g_{1}(\theta _{1}))\Big )+\cdots \end{aligned} \end{aligned}$$

**Fig. 3:**
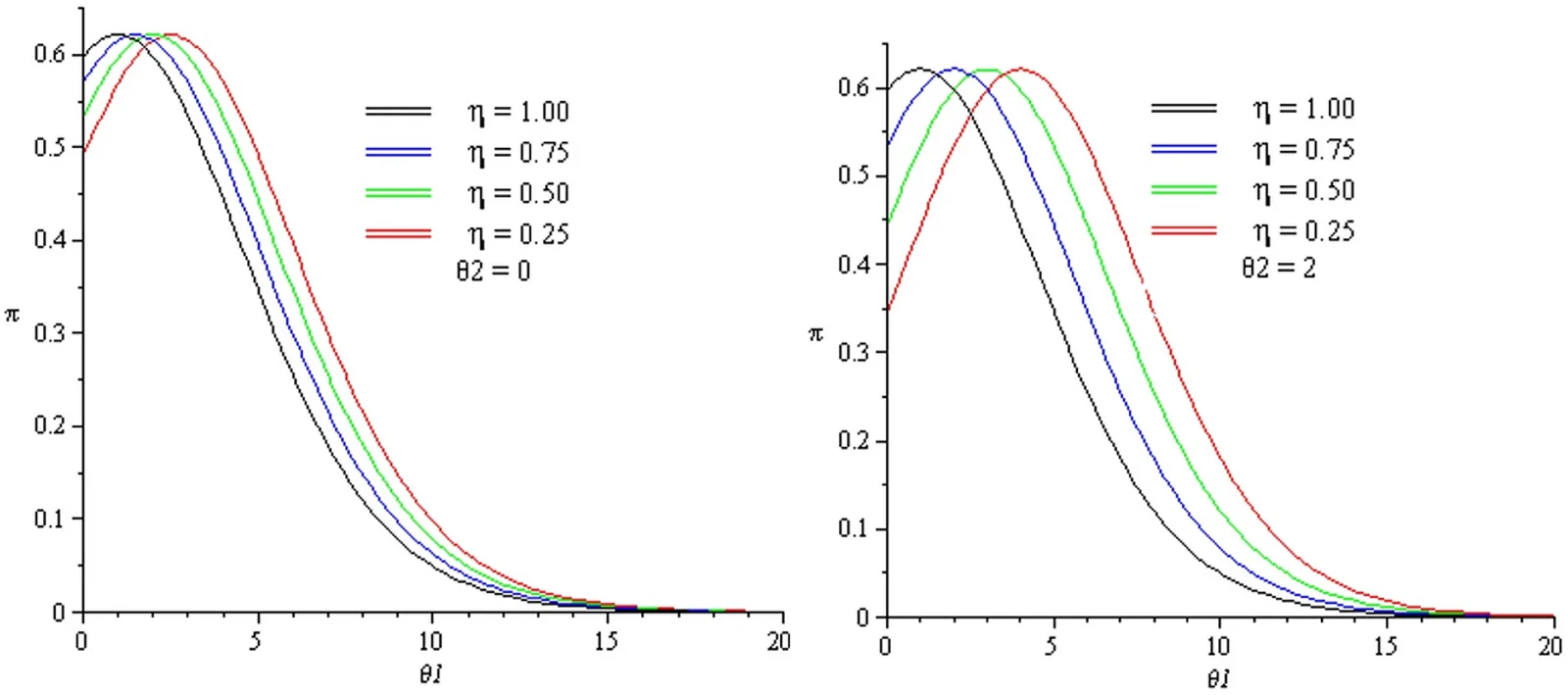
Yang RPSM curves of (41) with *λ =0.001*, *λ '=-1*, $\theta _{2}=0$, and $\theta _{2}=2$.

**Fig. 4:**
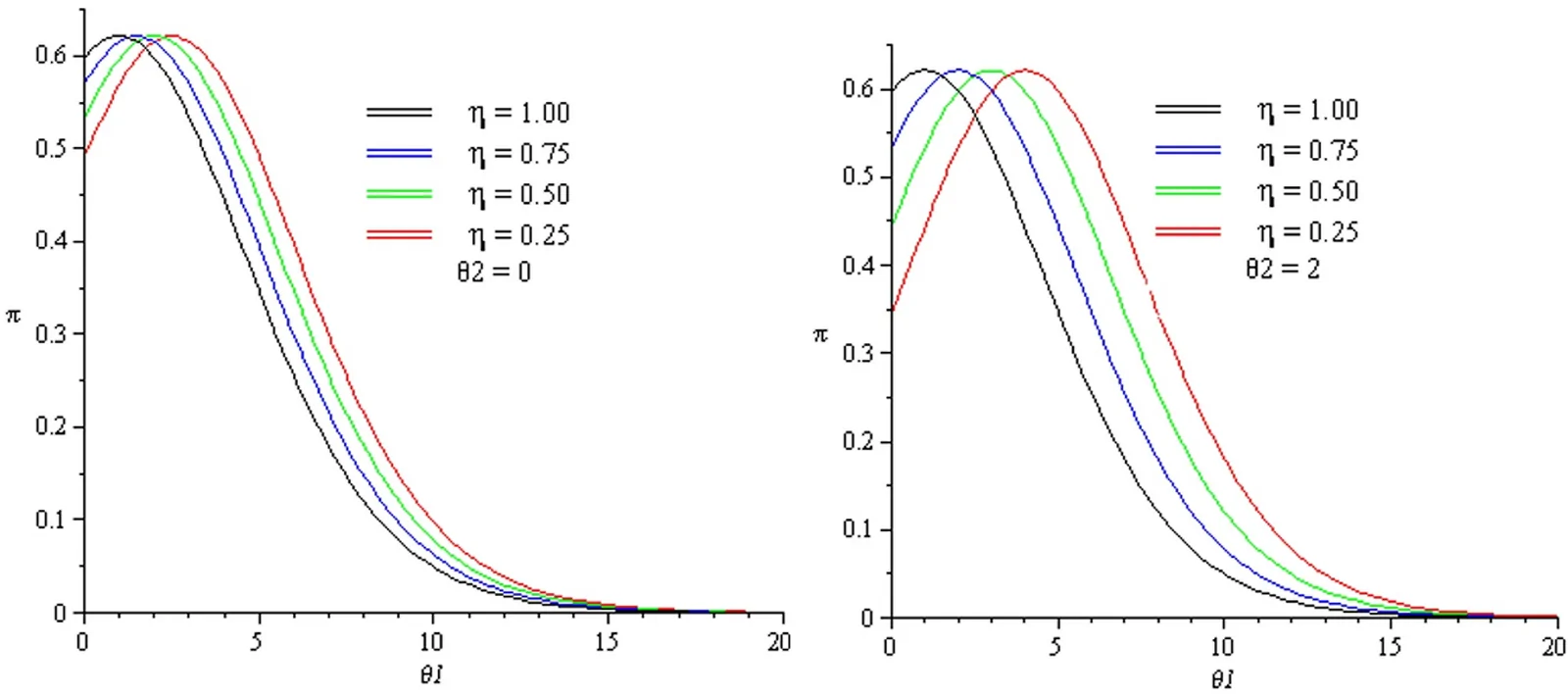
Yang RPSM curves of (41) with *λ =0.001*, *λ '=-1*, $\theta _{2}=4$, and $\theta _{2}=6.$.

**Fig. 5:**
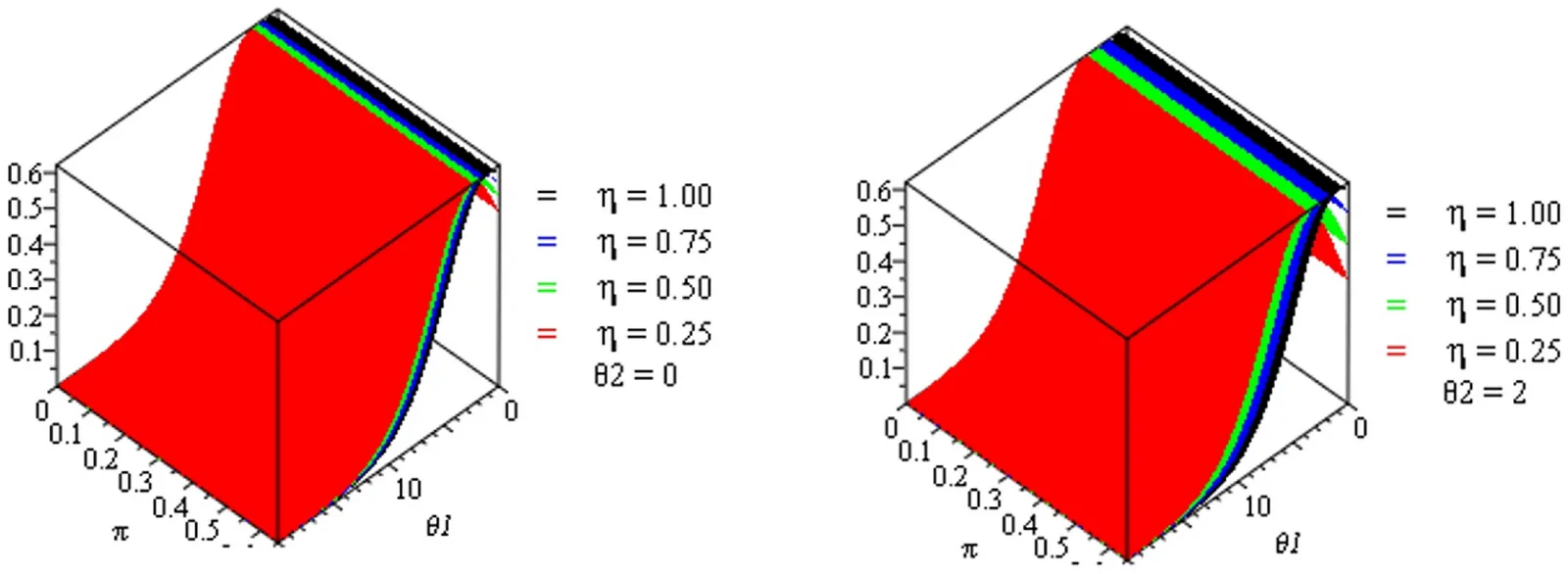
Plots of Yang RPSM solutions with *λ =0.001*, *λ '=-1*, $\theta _{2}=0$, and $\theta _{2}=2$.

**Fig. 6:**
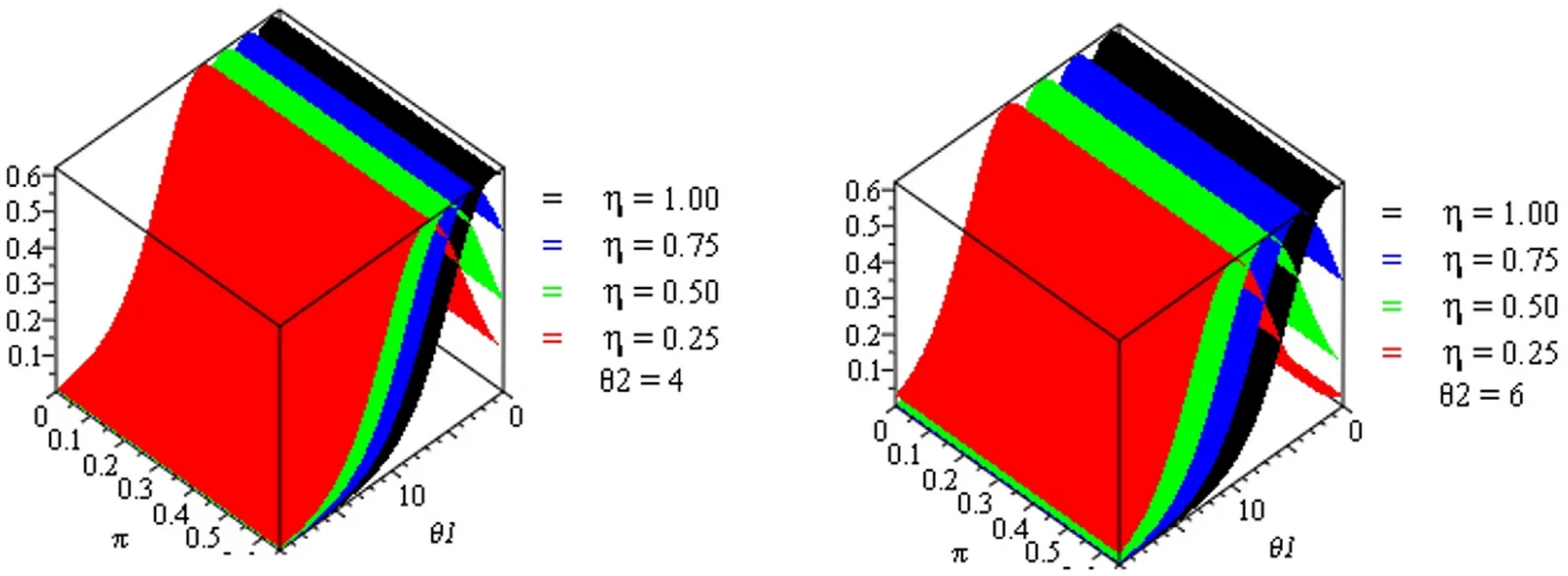
Plots of Yang RPSM solutions with *λ =0.001*, *λ '=-1*, $\theta _{2}=4$, and $\theta _{2}=6$.

## Numerical discussion

To rigorously assess convergence, we study the behavior of the Yang RPSM solution as the truncation order *n* increases. For a fixed set of parameters, we compute the absolute error between successive truncations and compare the convergence rate with NTDT and standard RPSM. This analysis provides direct insight into how rapidly each method approaches the limiting solution. In Tables 1, 2 and 3, we calculate some approximate solutions of (41) for different values of *η*. Figures 3, 4, 5 and 6 demonstrate the behavior of the solutions of (41). In Table 1, we provide comparisons of the absolute error (AE) at *λ =0.001*, *λ '=-1*, *η =1* and $\theta _{1}=10$ with some solutions of (41) obtained by the RPSM^42^ and the HAT in^36^. In Table 2, we compare the approximate solutions of (41) obtained using the Yang RPSM at *λ =0.001*, *λ '=-1*, $\theta _{1}=10$ and various values *η*, $\theta _{2}$ with some solutions obtained by RPSM in^42^ and the HAT in^36^. Figure 3 presents curves of the approximate solutions of (41) for *λ =0.001*, *λ '=-1*, $\theta _{2}=0$, $\theta _{2}=2$, and $\theta _{2}=4$. Figure 4 presents curves of the approximate solutions of (41) for *λ =0.001*, *λ '=-1*, $\theta _{2}=4$, $\theta _{2}=6$, and $\theta _{2}=4$. Figure 5 shows surface plots of Yang RPSM solutions with different values of *λ*, *λ '*, *η*, $\theta _{2}=0$, and $\theta _{2}=2$. Figure 6 shows surface plots of Yang RPSM solutions with different values of *λ*, *λ '*, *η*, $\theta _{2}=4$, and $\theta _{2}=6$. In these tables and graphs, we illustrate the applicability and accuracy of the Yang RPSM. The graphs and tables presented above highlight the accuracy and efficiency of Yang RPSM. The tables provide comparisons with some techniques, while the figures illustrate the symmetry and consistency in the graphical patterns of the three derivatives. The results demonstrate that Yang RPSM produces approximate solutions closely matching both exact and numerical solutions, confirming the method’s reliability. Its systematic and straightforward approach eliminates the need for linearization, discretization, or perturbation, thereby simplifying implementation. Furthermore, Yang RPSM generates solutions as rapidly convergent series, ensuring stability and high accuracy. A major advantage of Yang RPSM is its versatility, as it applies to both linear and nonlinear FDEs with varying fractional orders. Computationally, it is more efficient than many traditional numerical methods, requiring fewer calculations while maintaining comparable accuracy. Moreover, the method inherently incorporates fractional derivatives and integral operators, making it well-suited for modern fractional models. Finally, the series-based solutions allow flexible control over accuracy, as additional terms can be included to refine the approximation, achieving an effective balance between precision and computational cost. We observed that the Yang RPSM offers several advantages over the Laplace RPSM. Because the Yang transform has a simpler inversion structure and avoids the singular kernel issues common in Laplace based fractional transforms, the Yang RPSM produces cleaner residual formulations and requires fewer computational steps. It also demonstrates faster convergence for fractional orders and yields higher accuracy, as shown by smaller absolute errors in the numerical tables of the paper. The Laplace RPSM remains effective for integer-order problems, but it becomes more cumbersome for nonlinear fractional PDEs with high order derivatives due to complex transform rules and heavier symbolic manipulation. In contrast, the Yang RPSM handles the 5th-order dispersion and nonlinear terms of the MCFKE more efficiently and more stably. Therefore, based on analytical simplicity, convergence speed, computational efficiency, and numerical accuracy, Yang RPSM is the better method for solving the fractional Kawahara equation and similar FPDEs.

**Table 3:** Convergence comparison with respect to series order *n*.

Series order *n*	Yang RPSM	NTDT	RPSM
2	$1.2\times 10^{-6}$	$4.8\times 10^{-4}$	$4.8\times 10^{-4}$
4	$3.6\times 10^{-9}$	$2.1\times 10^{-6}$	$2.1\times 10^{-6}$
6	$7.4\times 10^{-12}$	$9.5\times 10^{-9}$	$9.5\times 10^{-9}$
8	$1.1\times 10^{-14}$	$3.8\times 10^{-11}$	$3.8\times 10^{-11}$

At the operator level, the distinction between the Yang RPSM and existing residual based methods lies in how the underlying transform maps the Caputo fractional derivative. This structure is directly compatible with the residual power series construction and allows the coefficients to be determined by enforcing the residual limit as $\upsilon \rightarrow 0$. In contrast, Laplace based RPSM formulations produce inverse power expansions in the Laplace variable, which introduce algebraic complexity in recursive coefficient identification and slow convergence. Similarly, NTDT decomposes the nonlinear operator but retains inverse transform operations at each iteration, increasing computational cost. Table 4 presented the operator level comparison of Yang RPSM with RPSM, Laplace RPSM, and NTDT. From a convergence and stability perspective, the Yang-based formulation avoids large inverse powers and yields a more stable recursion for the series coefficients. Since the residual enforcement relies on the limit $\upsilon \rightarrow 0$, rather than on iterative inversion, the method exhibits improved numerical robustness and reduced sensitivity to truncation order. Consequently, Yang RPSM requires fewer series terms to achieve comparable accuracy. Thus, the improvement over Laplace RPSM, NTDT, and RPSM is not merely numerical but originates from a fundamentally different operator induced series structure.

**Table 4:** Operator level comparison of Yang RPSM with existing methods.

Method	Transform structure	Residual enforcement	Computational cost
RPSM	Time-domain series	Direct substitution	Moderate
Laplace RPSM	Inverse-power series	Inverse Laplace limits	High
NTDT	Decomposition + transform	Iterative inversion	High
Yang RPSM	MFPS in positive powers	Limit $\upsilon \rightarrow 0$	Low

## Conclusions

This study introduced the Yang RPSM to obtain accurate approximate solutions of the MCFKE (3). The method proved stable, rapidly convergent, and more accurate than NTDT and HAT. Through applications to Ca$^{2+}$ dynamics, the MCFKE (3) was shown to capture key biological features: fractional order *η* models memory and subdiffusion, while the nonlinear and dispersive terms governed by $(\lambda ,\lambda ')$ reproduce wave sharpening, attenuation, and realistic intracellular propagation. The numerical results in Figs. 1 and 2 confirm the effectiveness of combining the MCFKE (3) with the Yang RPSM for modeling complex biochemical wave phenomena. Future work may extend the method to multidimensional or coupled systems, incorporate spatial fractional operators, develop data driven parameter estimation for $(\eta ,\lambda ,\lambda ')$, and explore applications to other nonlinear dispersive processes in biology and physics. These directions will further enhance the applicability and impact of the Yang RPSM and the MCFKE framework.

## Data Availability

All data are included in this article.
